# Grape Canes from Typical Cultivars of Campania (Southern Italy) as a Source of High-Value Bioactive Compounds: Phenolic Profile, Antioxidant and Antimicrobial Activities

**DOI:** 10.3390/molecules26092746

**Published:** 2021-05-07

**Authors:** Giuseppe Squillaci, Carla Zannella, Virginia Carbone, Paola Minasi, Veronica Folliero, Debora Stelitano, Francesco La Cara, Massimiliano Galdiero, Gianluigi Franci, Alessandra Morana

**Affiliations:** 1 Research Institute on Terrestrial Ecosystems, National Research Council of Italy, Via Pietro Castellino 111, 80131 Naples, Italy; giuseppe.squillaci@iret.cnr.it (G.S.); francesco.lacara@cnr.it (F.L.C.); alessandra.morana@cnr.it (A.M.); 2 Department of Experimental Medicine, University of Campania “Luigi Vanvitelli”, Via Costantinopoli 16, 80138 Naples, Italy; carlazannella88@gmail.com (C.Z.); veronicafolliero88@gmail.com (V.F.); debora.stelitano@unicampania.it (D.S.); massimiliano.galdiero@unicampania.it (M.G.); 3 Proteomic and Biomolecular Mass Spectrometry Center, Institute of Food Sciences, National Research Council of Italy, Via Roma 64, 83100 Avellino, Italy; virginia.carbone@cnr.it (V.C.); paola.minasi@cnr.it (P.M.); 4 Department of Medicine, Surgery and Dentistry Scuola Medica Salernitana, University of Salerno, 84081 Salerno, Italy

**Keywords:** antioxidant, antiviral activity, antimicrobial activity, grape cane, herpes simplex virus, phenolic compounds, Aglianico, Fiano, Greco, HPLC-MS

## Abstract

The purpose of the current study was to determine the phenolic composition, antioxidant, and antimicrobial activities in grape cane extracts from typical cultivars of Southern Italy. Aqueous extracts at different pHs (1–13) were prepared from “Aglianico”, “Fiano”, and “Greco” grape canes. The results demonstrated that an alkaline pH (13.00) produced the best polyphenol-rich extracts, as the total phenolic content was more than double when compared to the respective extracts prepared at pH 1.00. “Greco” grape canes gave the highest quantity of phenolic compounds at each pH, ranging from 42.7 ± 0.4 to 104.3 ± 3.0 mg Gallic Acid Equivalents (GAE)/g Dry Extract (DE) from pH 1.00 to 13.00. The Radical Scavenging Activity (RSA) and the Ferric Reducing Antioxidant Power (FRAP) were measured. The highest antioxidant activity was showed by “Greco” extract at pH 7.00. Seventy-five compounds were identified in the extracts by HPLC-MS with six of them described for the first time in grape canes. Procyanidins were highly abundant in extracts at pH 7.00, whereas stilbenoids were the most represented compounds at pH 13.00. Very strong antiviral activity against herpes simplex viruses was recorded for the extracts at pH 7.00 and 13.00 that were active in the early stages of infection by acting directly against the viral particles. The overall results suggest that grape canes, currently underutilized, can be usefully valorised by providing active extracts to use as antioxidant and antiviral agents.

## 1. Introduction

Interest in the exploitation of agro-industrial wastes as natural sources for the production of high added-value compounds is constantly growing. The circular economy model proposes the use of materials that were traditionally considered waste, as a resource [1]. 

Agricultural and industrial processing activities produce large amounts of liquid and solid wastes that can be conveniently utilised before disposal. Usually, these residues are burned or used for composting, even though they still contain valuable bioactive molecules that can be used for a number purposes in a range of sectors [2,3].

The wine production process generates a huge quantity of solid residues (skin, cane, stalk, and seed) whose management and disposal presents environmental issues. Several alternatives to the traditional disposal have been investigated, such as composting or removal of heavy metals from aqueous solutions [4,5] although wine residues are rich in bioactive compounds that can be recovered by suitable processes to provide valued products [6].

Vine tree (*Vitis vinifera* L.), belonging to the family of Vitaceae, is one of the fruit crops more broadly grown in many areas of the world. In 2020, about 7,231,542 tons of grapes were produced in Italy (652,451 ha of dedicated surface area). In Campania (South Italy) 25,562 ha are dedicated to this cultivation. “Aglianico” (red wine), “Fiano di Avellino” and “Greco di Tufo” (white wines hereafter referred to as “Fiano” and “Greco”), typical cultivars of the territory, produce excellent quality wines [7].

Grape cane is the main solid waste from vineyards; it is generated during the pruning process and about 1.4/2.0 tons/ha of this lignocellulosic residue are produced annually in a restricted time period [8]. Based on this assumption, about 35,787/51,124 tons/year of grape canes are produced in Campania, which are dumped in the fields to be used as compost or burned. Currently, few possible applications for this residue are reported; some examples include as a precursor of activated carbon or raw material for the production of biosurfactants [9,10]. Conversely, the exploitation of grape canes as a novel and cheap source of bioactive compounds, such as polyphenols, is poorly investigated, and the potential of grape cane extracts as multifunctional active ingredients in cosmetic formulations for skin care was very recently described [11]. These molecules, thanks to their well-known strong antioxidant power, are of significant interest for use in the food, cosmetic and pharmaceutical industries [12]. Furthermore, they possess anti-inflammatory and anti-cancer properties [13].

In recent years, some studies have shown the potential of the phenolic compounds from grape waste as antimicrobial agents [14,15]. Oliveira et al. [15] estimated the antimicrobial activity of pomace extracts from different grape varieties on several bacterial strains. The extracts were more effective against *Bacillus cereus* and *Staphylococcus aureus* (*S. aureus*) and less active against *Escherichia coli* (*E. coli*) and *Pseudomonas aeruginosa*. Little is known about the antimicrobial properties of grape canes. The study of Moreira et al. [16] refers to grape cane extracts active against Gram- (*E. coli* ESA 37 Cephalosporins resistant) and Gram+ (*Streptococcus mitis*, *S. mitis*) bacteria. Ohmic heating polyphenolic extracts from grape canes were tested against five food pathogenic filamentous fungi (*Alternaria* sp., *Cladosporium cladosporioides*, *Phoma violacea*, *Penicillium italicum* and *P. expansum*) showing greater activity after 96 h of exposure at 1 mg/mL [17]. However, although some investigations have reported on the antibacterial activity of grape canes, none of them have described a possible antiviral effect.

Presently, a major public health concern is the emergence of multidrug-resistant pathogens due to the overconsumption of medicinal drugs. This is not a problem restricted to bacteria, but it involves all microbes able to resist the common therapies. It has been estimated that antimicrobial-resistant infections lead to at least 700,000 deaths annually [18]. 

The herpes simplex virus (HSV) infection represents one of the most common infectious diseases in humans. The virus can be classified into two types: herpes simplex virus type 1 (HSV-1) and type 2 (HSV-2) [19]. It has been estimated that 3.7 billion people under the age of 50 (66.6% of world population) are infected by HSV-1 [20]; moreover, the World Health Organization (WHO) has observed that 491.5 million people aged 15 to 49 years (13.2%) have HSV-2 infection. Many different drugs are currently used for HSV treatment, such as acyclovir, penciclovir, and other guanine analogues. However, even for herpes infections, the emergence of drug resistance is commonly observed, to which the inability to eradicate latent infections is added [21].

In this context, scientific interest has been moving in developing alternative therapies based on natural drugs, in particular plant-derived compounds and a great emphasis has been focusing on agricultural by-products that are usually wasted [22,23]. Furthermore, an increasing number of scientific papers and pharmaceutical products are based on natural miscellaneous compounds that act in a synergic way with a double vantage: they are less toxic compared to a single synthetic compound and they are more active [24].

Generally, free polyphenols along with glycosides and oligomers can be readily extracted from agro-food industrial waste and by-products by means of organic solvents [25]. However, these processes have an undesirable environmental impact and for this reason, intensive research is focused on new sustainable methods for the recovery of phenolic compounds from agro-food residues. In the current study, a sustainable extraction for the retrieval of phenolic compounds from grape canes belonging to “Aglianico”, “Fiano” and “Greco” Campania cultivars using water as an extraction solvent at different pHs was applied. Total phenol, flavonoid, *ortho*-diphenol and tannin contents were determined in the extracts, and the antioxidant activity was also evaluated. Moreover, a comprehensive quali-quantitative study of the phenolic molecules present in selected extracts was carried out by high-performance liquid chromatography coupled with multistage ion trap mass spectrometry (HPLC/ITMSn) and UV detection (HPLC-UV). Finally, the antimicrobial activity of the extracts against bacteria, viruses and fungi causing human infections was also investigated. 

## 2. Results and Discussion 

### 2.1. Grape Canes Extraction

Powdered grape canes were dried in order to remove the water content prior to the extraction. The moisture contents were 15.4% for “Aglianico”, 22.0% for “Fiano”, and 19.3% for “Greco”. As the interest in using eco-compatible solvents has increased in recent times due to environmental and health and safety concerns, the effect of water as a solvent for the extraction of polyphenols from grape canes was investigated due to its non-toxicity and low environmental impact [26]. The pH effect during the extraction process (1.00–13.00) was considered in this set of experiments to investigate its influence on the yield, and on the qualitative and quantitative composition of the extracts from the different cultivars. The influence of time and temperature was previously studied [27]. The extraction yields ranged from 18.1 (pH 1.00) to 25.0% (pH 13.00) for “Aglianico”, from 17.6 (pH 1.00) to 22.4% (pH 13.00) for “Fiano”, and from 17.7 (pH 1.00) to 23.8% (pH 13.00) for “Greco”. Our values were corroborated by those reported by Zhang et al. [12] who carried out an extraction from the grape canes of 11 different cultivars. Although the extractions were performed in an acidified methanol/water mixture (80:20 *v*/*v*) for 24 h at 20 °C, the yields were between 14.9% and 26% (Junzi and Cabernet Sauvignon cultivars, respectively). Under our experimental conditions, the extract containing the highest quantity of phenolic compounds was obtained from the “Greco” cultivar at pH 13.00 (104.3 ± 3.0 mg Gallic Acid Equivalents, GAE/g Dry Extract, DE) whereas the extract with less polyphenols was obtained from “Fiano” at pH 1.00 (30.3 ± 1.5 mg GAE/g DE).

### 2.2. Characterization of Grape Cane Extracts

**Figure 1:**
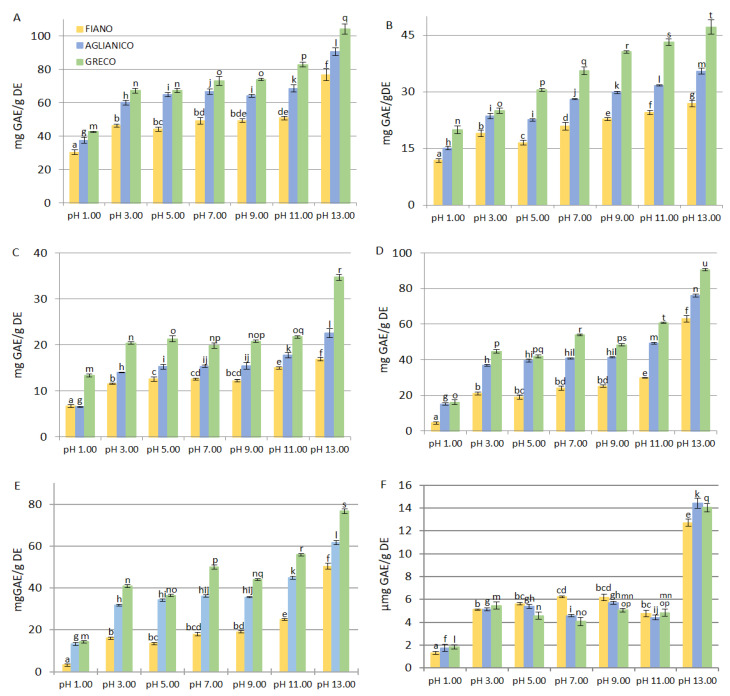
Total phenols (**A**), *ortho*-diphenols (**B**), flavonoids (**C**), total tannins (**D**), condensed tannins (**E**), and hydrolysable tannins (**F**) in grape cane extracts from *Vitis vinifera* cultivars “Aglianico, “Fiano” and “Greco”. All determinations were performed in triplicate and results were expressed as mean ± SD. Bars with different letters denote significant differences at *p* < 0.05. (**A**: a-f, g-l, m-q; **B**: a-g, h-m, n-t; **C**: a-f, g-l, m-r; **D**: a-f, g-n, o-u; **E**: a-f, g-l, m-s; **F**: a-e, f-k, l-q).

Total phenol, *ortho*-diphenol, flavonoid and tannin contents in the grape cane extracts are reported in Figure 1. Different amounts were obtained from the three cultivars also depending on the pHs. The extracts from “Greco” and “Fiano” contained the highest and the lowest amount of total phenolic compounds at every pH, respectively; furthermore, the high pH favoured the phenolic compounds extraction (Figure 1A). In detail, 30.3 ± 1.5 (“Fiano”), 37.4 ± 1.7 (“Aglianico”), and 42.7 ± 0.4 (“Greco”) mg GAE/g DE at pH 1.00, and 76.8 ± 3.5 (“Fiano”), 90.7 ± 2.4 (“Aglianico”), and 104.3 ± 3.0 (“Greco”) mg GAE/g DE at pH 13.00 were measured. As shown in Figure 1A, extraction pHs from 3.00 to 9.00 and from 3.00 to 11.00 did not significantly affect the total phenol content in the cultivars “Aglianico” and “Fiano”, respectively (*p* > 0.05). Rajha et al. [28] studied the effect of the NaOH concentration on the extraction of phenolic compounds from grape canes (Syrah variety), and their results confirmed that high pH enhances polyphenols recovery; in fact, the total phenols extracted accounted for 0.3 and 4.8 mg GAE/g dry matter for 0 and 1 M NaOH, respectively. 

As compounds with a catechol group exhibit high antioxidant activity due to the ability to stabilize the phenoxyl free radicals through the formation of intramolecular hydrogen bonds between the radical oxygen and the adjacent hydroxyl group [29], the *ortho*-diphenol amount was estimated (Figure 1B). The concentration ranged from 11.81 ± 0.50 (“Fiano” pH 1.00) to 47.12 ± 1.89 (“Greco” pH 13.00) mg GAE/g DE. Considerable variability was observed for the three cultivars. Within the same cultivar, the *ortho*-diphenol content was significantly different at all pH values (*p* < 0.05). Flavonoids and total tannins followed the same trend, as shown by the total phenols and *ortho*-diphenols. In general, their quantity increased from acid to alkaline pH and the total content was in the order: “Fiano”<“Aglianico”<“Greco”. At the extreme pH values, the following flavonoid content was measured: 6.75 ± 0.32 mg GAE/g DE (pH 1.00) and 16.88 ± 0.36 mg GAE/g DE (pH 13.00) for “Fiano” (*p* < 0.001), 6.83 ± 0.19 mg GAE/g DE (pH 1.00) and 22.56 ± 0.94 mg GAE/g DE (pH 13.00) for “Aglianico”(*p* < 0.001), 13.35 ± 0.35 mg GAE/g DE (pH 1.00) and 34.70 ± 0.59 mg GAE/g DE (pH 13.00) for “Greco” (*p* < 0.001) (Figure 1C). The total tannin content was extremely low in “Fiano” at pH 1.00 (4.54 ± 0.64 mg GAE/g DE), but it increased 4.6-fold at pH 3.00 (21.14 ± 0.87 mg GAE/g DE (*p* < 0.001) reaching 63.05 ± 1.74 mg GAE/g DE at pH 13.00 (*p* < 0.001) (Figure 1D). At this pH, the values for “Aglianico” and “Greco” were 76.18 ± 0.80 and 90.67 ± 0.68 mg GAE/g DE, respectively. Total tannins were the main class of polyphenols present in the extracts. As an example, they accounted for 45.64%, 61.27%, and 66.41% of the total phenols in extracts at pH 3.00 from “Fiano”, “Aglianico” and “Greco” cultivars, respectively. This percentage gradually increased, until reaching 82.11%, 84.00%, and 86.92% for “Fiano”, “Aglianico” and “Greco” at pH 13.00, respectively (*p* < 0.001). Total tannins are composed of condensed tannins (or proanthocyanidins) and hydrolysable tannins. Condensed tannins are oligomers or polymers of flavonoid and can reach high molecular weights, whereas hydrolysable tannins are formed by a core constituted by a polyol; the most common is glucose, totally or partially esterified by gallic acid or ellagic acid. 

The yields in condensed tannins were similar to the yields in total tannins. In fact, their amount increased from pH 1.00 to pH 13.00 in all cultivars (*p* < 0.001). “Fiano” contained the smallest quantity, whereas “Greco” was the richest at each extraction pH (Figure 1E). Different conditions were verified for the hydrolysable tannins; in fact, “Fiano” extracts at pH 5.00, 7.00, and 9.00 showed the highest content compared to the other two cultivars extracted at the same pH. Moreover, the amount measured at pH 3.00 and 11.00 was comparable to the amount from the other cultivars: “Fiano” 5.10 ± 0.04, “Aglianico” 5.14 ± 0.13, “Greco” 5.45 ± 0.30 mg GAE/g DE, and “Fiano” 4.78 ± 0.29, “Aglianico” 4.42 ± 0.19, “Greco” 4.83 ± 0.32 mg GAE/g DE, respectively (Figure 1F). The analysis of variance revealed that flavonoid and tannin contents were significatively affected by the extreme pHs of extraction (*p* < 0.001), while no significant differences were observed at intermediate pH values (*p* > 0.05).

The multitude of different results that natural extracts of different origins may offer, combined with the extraction processes that can vary for the operating parameters (extraction time, solvent, solid/liquid ratio, biomass concentration particle size, etc.), makes the comparison among extraction yields and bioactive compounds composition from different research groups quite difficult.

The overall findings allowed to attest differences in the phenolic content among the three selected cultivars. This was in agreement with studies on grapes and grape wastes from diverse cultivars where the bioactive molecules content may vary in relation to several factors, such as cultivar, vineyards management, and growing conditions [30].

The study of Zhang et al. [12] on the phenolic composition of grape canes from 11 Chinese varieties (five *V. vinifera* cultivars and six Chinese wild grapes) confirmed the great variability due to the influence of the cultivar and the environmental factors. The total phenolic content in the extracts prepared by acidified methanol at 20 °C was comprised between 76.4 (Chardonnay) to 224.5 (Junzi) mg GAE/g DE, whereas the total flavonoid content varied from 33.1 (Victoria Blanc) to 146.6 (Junzi) mg Quercetin Equivalent (QE)/g DE. Grape canes from native grapes cultivated in West Azerbaijan, extracted using acidified methanol in an ultrasound bath, showed variability of the phenolic and flavonoid contents. The phenolic content ranged from 175 (Hosseini) to 250 (Ghara Shani) mg GAE/g dry matter and flavonoid content ranged from 160 (Hosseini) to 219 mg QE/g dry matter (Ghara Shani) [31].

Great variability was also measured in grape skin other than grape canes. Differences in the extracts from seven red and seven white grape varieties from Croatia attested that cultivar and pedoclimatic conditions may influence the composition of the bioactive compounds in the whole tree. Total flavonoids were comprised between 389 (Medna) and 1182 (Zlatarica) mg GAE/kg fresh matter, and between 400 (Lasin) and 2594 (Rudežša) mg GAE/kg fresh matter, for white and red varieties, respectively [32].

Our findings did not reveal significant differences at intermediate pH values within the same cultivar (*p* > 0.05). Therefore, the extracts prepared at pH 1.00, 7.00, and 13.00 (extremes and central values) were selected for further analyses.

### 2.3. Identification and Quantification of Phenolic Compounds

**Figure 2:**
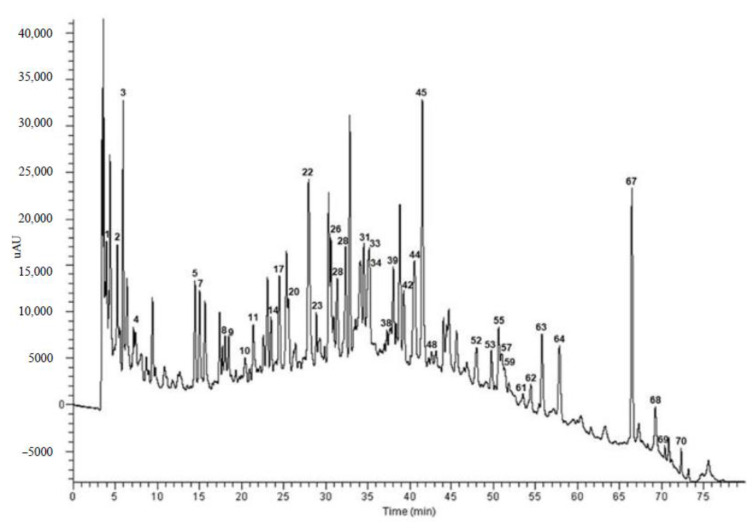
HPLC-UV chromatogram recorded at 280 nm of phenolic compounds present in grape cane extract at pH 1.00 from “Aglianico” cultivar. For chromatographic conditions, see Section 3.3.6. For peak assignments, see Table 1.

The separation, identification and quantification of phenolic compounds contained in selected grape cane extracts (pH 1.00, 7.00, and 13.00) were carried out by HPLC/ESI-ITMS^n^ and HPLC-UV analyses. HPLC-UV chromatogram of phenolic compounds present in grape cane extract from “Aglianico” cultivar (pH 1.00) is shown in Figure 2. 

Overall, the performed analysis led to tentatively identify 75 different compounds on the basis of their pseudomolecular [M-H]^−^ ions, together with the interpretation of their collision-induced dissociation (CID) fragments. When authentic standards were available, identification was conducted by comparing retention times and MSn fragmentation spectra with those of standards. The classes of phenolic compounds detected agreed with those already reported in previous studies on the phenolic profile of grape canes [16,33] and included phenolic acids, flavanols, flavonols, flavanonols, flavanones, and stilbenoids. The list of compounds identified in the selected grape cane extracts and their quantification are reported in Table 1 and Table 2. The phenolic composition was qualitatively and quantitatively affected by the extraction pH: the glycosylated forms were predominant at pH 1.00, procyanidin oligomers were very abundant in extracts at pH 7.00, and stilbenoids were most represented at pH 13.00.

The amount of the stilbenoid resveratrol (peak 67) increased from pH 1.00 to pH 13.00 varying from 220.43 ± 16.84 to 1741.01 ± 62.45 µg/g DE (*p* < 0.001), from 51.51 ± 3.68 to 580.42 ± 0.33 µg/g DE (*p* < 0.01), and from 136.79 ± 43.31 to 1398.06 ± 23.58 µg/g DE (*p* < 0.001) for “Aglianico”, “Fiano”, and “Greco”, respectively. The resveratrol dimer, ε-viniferin (peak 70), was particularly abundant in the alkaline extracts with 1657.60 ± 18.80 µg equivalent/g DE (“Aglianico”), 1617.56 ± 199.55 µg equivalent/g DE (“Fiano”), and 1543.28 ± 97.67 µg equivalent/g DE (“Greco”) in contrast to the acid and neutral extracts.

The grape cane extract from “Aglianico” was the richest in polyphenol species at each pH, since 42, 48 and 33 compounds were identified at pH 1.00, 7.00, and 13.00, respectively. On the contrary, in the extracts from “Fiano” the lowest number of polyphenolic compounds was identified: 27, 37, and 18 at pH 1.00, 7.00, and 13.00, respectively. 

Tartaric acid (peak 1), the hydroxybenzoic acid derivatives protocatechuic acid glucoside (peaks 11 and 17), syringic acid hexoside (peak 20), the hydroxycinnamic acid derivative caftaric acid (peak 22), the flavanol catechin (peak 31), the stilbenoids resveratrol-*C*-glucoside (peak 45), resveratrol (peak 67) and ε-viniferin (peak 70) were identified in all the extracts, together with afzelechin hexoside (peak 8) and viniferol E (peak 68). To the best of our knowledge, afzelechin hexoside and viniferol E were detected for the first time in grape canes. Viniferol E was previously isolated in different *Vitis vinifera* parts, such as the roots of cultivar “Kyohou” and grape stems of eight French cultivars located in the Loire Valley region [34,35].

Ellagic acid (peak 54) and its derivative ellagic acid pentoside (peak 51) were not detected at acidic pHs. More precisely, ellagic acid was identified in “Aglianico” at pH 7.00 and in all the extracts at pH 13.00, whereas ellagic acid pentoside was detected in all extracts at pH 7.00 and 13.00 ranging from 39.64 ± 10.26 to 174.86 ± 0.66 µg equivalent/g DE and from 95.75 ± 18.77 to 354.44 ± 3.80 µg equivalent/g DE, respectively. On the contrary, the hydroxycinnamic acid derivative rosmarinic acid hexoside (peak 42) was not detected at pH 13.00 and its highest amount was measured in “Fiano” extract at pH 7.00 (3005.53 ± 128.41 µg equivalent/g DE). It is noteworthy that the non-glycosylated form of rosmarinic acid was previously found in winery pomace from red and white grapes [36], but the presence of rosmarinic acid hexoside in grape canes is here reported for the first time. 

HPLC/ESI-ITMS^n^ analyses of the acid extracts showed in peak 53 a compound with pseudomolecular ion [M-H]^−^ at *m*/*z* 419 in the mass spectrum. In the MS2 spectrum, the following fragmentation pattern was obtained: *m*/*z* 287, corresponding to the deprotonated ion of dihydrokaempferol (or eriodictyol) after the loss of a pentose moiety [M-H-132], and *m*/*z* 269, due to the successive loss of water. 

Therefore, the compound eluted in peak 53 was tentatively identified as dihydrokaempferol pentoside or eriodictyol pentoside. Dihydrokaempferol and eriodictyol were previously detected as aglycones and/or hexoside in *Vitis vinifera* by-products. To the best of our knowledge, this is the first report describing the presence of dihydrokaempferol pentoside or eriodictyol pentoside in grape canes. Only in the “Aglianico” grape cane extract prepared at pH 1.00, a second compound eluted at the same retention time of eriodictyol pentoside (or dihydrokaempferol pentoside) (peak 53) and was identified as flavonol rutin (quercetin-3-*O*-rutinoside). 

In acid and neutral extracts, the analysis by HPLC/ESI-ITMSn allowed us to detect, for the first time, the occurrence in grape canes of A-type procyanidin dimers, non-galloylated and digalloylated. Grape proanthocyanidins are essentially of B-type, but Passos et al. [37] reported evidence for the existence of galloylated A-type procyanidins in grape seeds. 

Interestingly, some phenolic compounds were distinctive of the cultivar “Aglianico” such as the hydroxycinnamic acid derivative coutaric acid (ester between coumaric acid and tartaric acid) (peak 34) and the aforementioned flavonol derivative rutin, both present in the acid extract, and the flavanone derivative naringenin-*O*-hexoside (peak 64), identified at all pH values in quantity comprised between 132.93 ± 3.35 (pH 1.00) and 275.35 ± 2.87 µg equivalent/g DE (pH 7.00). As far as we know, this is the first report describing the presence of naringenin-*O*-hexoside in grape cane extracts. As a matter of fact, only the aglycone naringenin and its disaccharide derivative naringin were previously detected in grape canes from eight varieties located in the Mendoza region, Argentina [30]. Finally, two of the three isomers of (epi)catechin-(epi)gallocatechin were exclusively detected in the “Aglianico” extract prepared at pH 7.00 (peaks 16 and 21). The third isomer was detected in the “Aglianico” and “Greco” extracts prepared at all pH values (peak 23), while all the isomers were absent in the extracts from “Fiano”.

### 2.4. Antioxidant Power

The antioxidant power of natural extracts is due to a mixture of several phytochemicals that can act with several and/or different mechanisms. According to this, two analyses based on diverse action mechanisms were used for determining the antioxidant capacity of the grape cane extracts: the Radical Scavenging Activity (RSA) and the Ferric Reducing Antioxidant Power (FRAP) assays.

The deleterious effects of free radicals in oxidative processes involving biological systems, foods, cosmetics, and pharmaceuticals are known. The prevention of the initiation step in the radical chain by scavenging reactive species such as free radicals is considered an important antioxidant mode of action. Thus, the antioxidant power of “Aglianico”, “Fiano” and “Greco” extracts at pH 1.00, 7.00 and 13.00 was investigated through the evaluation of the discoloration of DPPH∙, a free radical that accepts an electron or a hydrogen radical to become a stable molecule.

**Figure 3:**
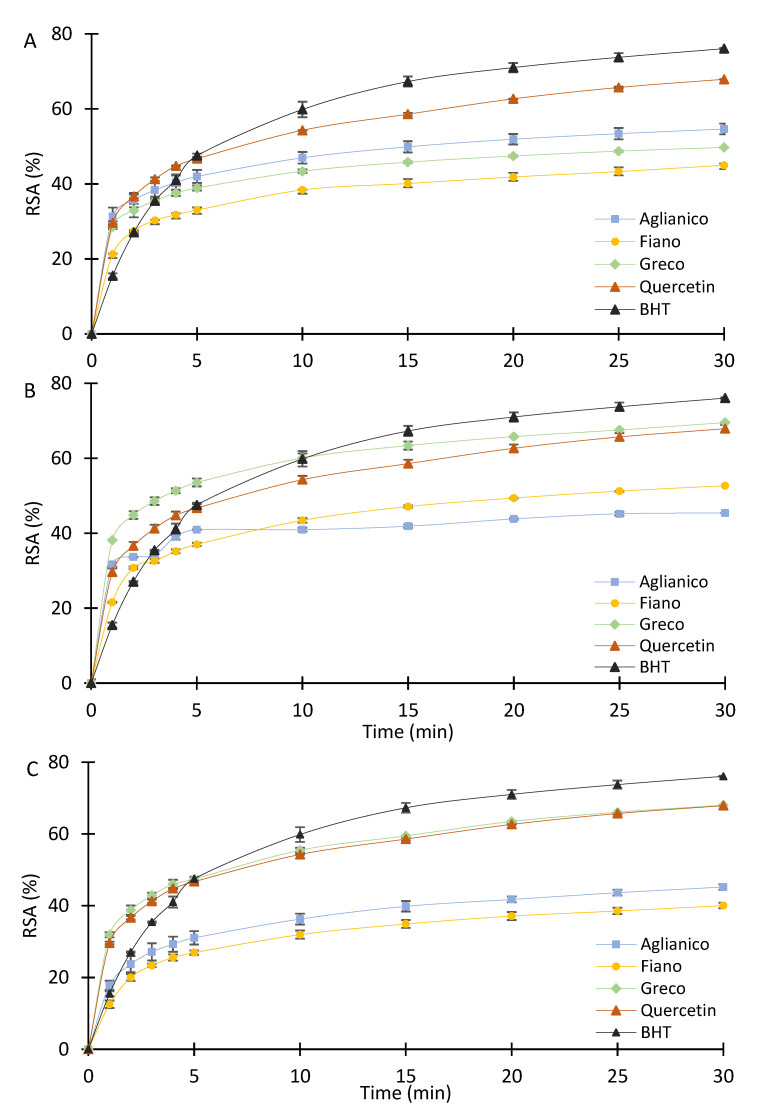
Free Radical Scavenging Activity of “Aglianico”, “Fiano”, and “Greco” grape cane extracts at pH 1.00 (**A**), 7.00 (**B**), 13.00 (**C**), and antioxidant reference compounds. DPPH assay was performed with 5 μg GAE of extracts or pure reference compounds as described in Section 3.4.1. All determinations were performed in triplicate and results were expressed as mean ± SD.

All the tested samples exhibited antioxidant activity, with values of RSA ranging from 40% ± 0.8 (“Fiano” pH 13.00) to 70% ± 0.11 (“Greco” pH 7.00) (Figure 3). RSA rapidly increased within the first minutes of assay, slowing down as the reaction time increased; nevertheless, differences among the extracts were measured according to the extraction pHs and to the cultivar. Among the extracts at pH 1.00, “Aglianico” exhibited the highest radical scavenging effect (55% ± 1.4) (Figure 3A). “Greco” grape canes provided the extracts with the highest RSA at pH 7.00 and 13.00 (70% ± 0.11 and 68% ± 0.12, respectively), while “Fiano” showed the best radical scavenging effect at pH 7.00 (53% ± 0.02) (Figure 3B,C). A comparison among the RSA of the extracts and the standards quercetin (Q) and butylatedhydroxytoluene (BHT) was carried out by using 5 μg GAE of each tested sample. Q and BHT were selected as reference compounds for the following reasons: Q is a natural antioxidant occurring in red wine, tea, berries, and other vegetables and fruits. This flavonoid has strong antioxidant power and is used in cosmetic formulations for its ability to neutralize free radicals. Furthermore, it is also a component of many nutritional supplements. BHT was chosen as representative of synthetic antioxidants, whose safety is currently discussed [38]. It is added as a preservative to foods rich in oils and fats to prevent their oxidation.

In the first five minutes of the assay, the “Greco” extract at pH 7.00 showed a value higher than the reference antioxidant compounds (“Greco” 54% ± 0.7, Q 47% ± 0.5, BHT 48% ± 0.6), and after 30 min, its RSA was still slightly higher than Q (70% ± 0.11 and 68% ± 0.06, respectively) (Figure 3B). Even at pH 13.00, “Greco” exhibited the best RSA, showing a value higher than BHT in the first 4 min of assay (46% ± 1.5 and 41% ± 1.5, respectively), and an antioxidant power equal to Q after 30 min (68%) (Figure 3C). DPPH assay is a widely used method for evaluating the free radical scavenging activity of natural compounds because of its stability, simplicity, and reproducibility. The grape cane extracts from six cultivars located in Iran were assayed by means of the DPPH method for the determination of their antioxidant power. The RSA measured after 30 min was quite similar among the cultivars, being comprised between 89.57% (Ghara Shira) and 94.63% (Ghara Shani) [31]. Although these values were higher than “Greco” at pH 7.00, it must be underlined that grape canes were extracted by acidified methanol, and alcoholic extracts differ for quantitative and qualitative aspects from aqueous extracts being the extraction of some phenolic compounds favoured in the first solvent.

In the present paper, it was decided to sacrifice the higher yield and representativeness of the phenolic compounds to the advantage of an extraction method that uses a non-toxic and environmentally friendly solvent. Nevertheless “Greco” extracts at pH 7.00 and 13.00 exhibited a significant radical scavenging activity.

The grape cane extracts showed antioxidant capacity when assayed by FRAP, attesting the ability of reacting with ferric ions. The highest FRAP values were measured in extracts at pH 7.00 with “Greco” having reached the value of 39.482 ± 1.287 μg Ascorbic Acid Equivalents (AAE)/mg DE. “Aglianico” and “Fiano” reached 32.058 ± 2.051 and 24.179 ± 0.681 μg AAE/mg DE, respectively.

**Figure 4:**
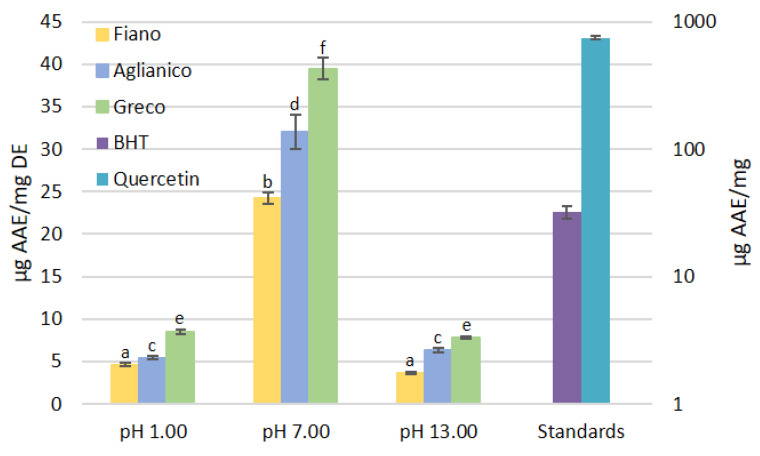
Ferric Reducing Antioxidant Power of “Aglianico”, “Fiano”, and “Greco” grape cane extracts at pH 1.00, 7.00, 13.00, and antioxidant reference compounds. The assay was performed with 1 mg of DE or 1 mg of pure reference compounds, as described in Section 3.4.2. All determinations were performed in triplicate and results were expressed as mean ± SD. Standards are shown in log scale due to quercetin’s high antioxidant activity. Bars with different letters denote significant differences at *p* < 0.001 (a-b, c-d, e-f).

Extracts at pH 1.00 and 13.00 were endowed with lower antioxidant power, ranging from 3.649 ± 0.131 (“Fiano” pH 13.00) to 8.439 ± 0.282 (“Greco” pH 1.00) μg AAE/mg DE. Only the “Greco” pH 7.00 was found to be more antioxidant than BHT used as standard. “Fiano” pH 7.00 and “Aglianico” pH 7.00, however, showed FRAP values comparable to those obtained with BHT, even if slightly lower. The extracts at pH 1.00 and 13.00 showed much lower FRAP values than BHT. Quercetin was found to be hugely more antioxidant than all extracts and BHT. For this reason, the standards are shown in logarithmic scale in Figure 4.

The results indicated that changes of pH in the extraction process from 1.00 to 7.00 lead to a statistically significant increase of the FRAP values (*p* < 0.001) within the same cultivar. The same level of significance was reached in the further increase of pH towards alkaline values (pH 13.00). In this case, the decrease of FRAP values obtained from extracts of the same cultivar (pH 7.00 and 13.00) was statistically significant (*p* < 0.001).

A comparison between the results here obtained and those reported by other groups is quite difficult, as in most cases, conventional extractions use alcoholic solvents. The typical variability of each cultivar must be added to the different extraction conditions, as demonstrated in the present work, where “Aglianico”, “Fiano” and “Greco” showed different FRAP values. In support of this, the work of Dorosh et al. [39] describes the valorization of grape canes from two Portuguese varieties (Touriga Nacional and Tinta Roriz) through ultrasound-assisted extraction carried out at different times (15, 30 and 60 min). FRAP values ranged from 5.2 to 8.9 mg AAE/g dry weight at 15 and 60 min, respectively, for Touriga Nacional, and from 12.4 to 20.1 mg AAE/g dry weight at 15 and 60 min, respectively, for Tinta Roriz.

The positive responses obtained with DPPH and FRAP assays attested the capacity of the extracts to act as antioxidants through the transfer of electrons to radical species (DPPH) or to ions with high oxidation number (ferric ions), highlighting that “Greco” grape canes, extracted at pH 7.00, can be considered as a valuable resource for the production of natural antioxidants to use in several fields such as food, pharmaceutical and cosmetic industries.

The results regarding the antioxidant activity can be further investigated in the future by coupling the traditional antioxidant assays with more recent techniques such as cyclic voltammetry [40].

### 2.5. Cytotoxicity Evaluation

**Figure 5:**
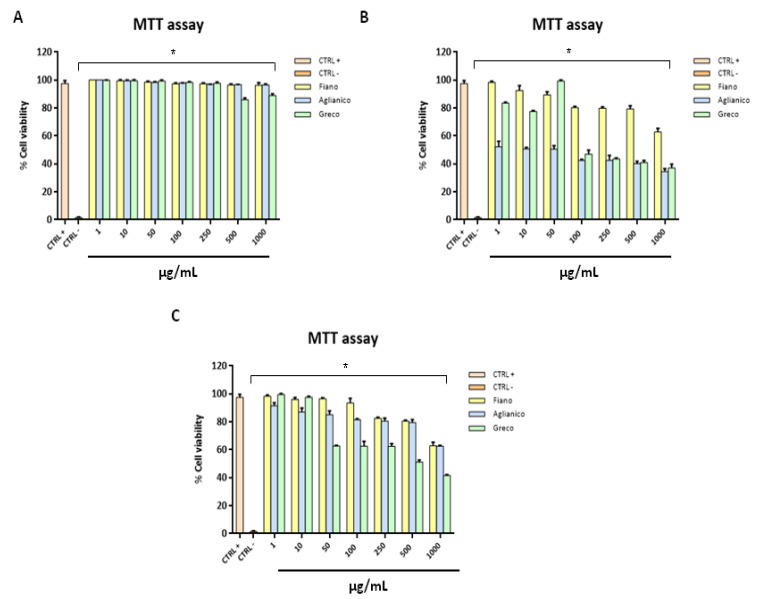
Cytotoxicity evaluation at different extraction pH. Cytotoxicity was assessed via MTT assay after 24 h post-treatment at pH 7.00 (**A**), 1.00 (**B**), and 13.00 (**C**). Statistical differences were evaluated via two-way ANOVA; a value of *p* ≤ 0.05 was considered significant, with * *p* ≤ 0.0001.

The cytotoxicity was evaluated in vitro through the metabolic activity of viable cells via 3-(4,5-dimethylthiazol-2-yl)-2,5-diphenyl-2H-tetrazolium bromide (MTT) assay [41]. Extracts at different pHs were analysed: none of the extracts at pH 7.00 were toxic for cell monolayers after 24 h, even at the highest concentration (Figure 5A).

On the contrary, a considerable reduction of viability was observed with some extracts at pH 1.00, and less marked with extracts at pH 13.00. Setting 50% of cell viability as a threshold line (CC50), “Aglianico” grape cane extract at pH 1.00 showed the highest cytotoxicity up to the lowest concentration tested (1 µg/mL). Furthermore, “Greco” at pH 1.00 was cytotoxic, exhibiting a CC50 at 50 µg/mL, while “Fiano” at pH 1.00 did not cause cellular death except at 1000 µg/mL (Figure 5B). The cytotoxicity decreased considerably treating cell monolayers with extracts at pH 13.00. In fact, only “Greco” exhibited a significant toxicity at concentrations of 500 and 1000 µg/mL (Figure 5C). Data were statistically significant with a p-value less than 0.05 (*p* ≤ 0.05). The general results showed that the acidic extraction pH was critical for the cell viability, in particular with “Aglianico” and “Greco” grape canes. It could be justified since when the pH of the extraction varies, the corresponding phenolic composition also clearly changes both in qualitative and quantitative terms, as demonstrated earlier in our work. Consequently, the following antimicrobial assays were performed with extracts prepared at pH 7.00 and 13.00.

### 2.6. Antimicrobial Activity

In order to evaluate the antimicrobial activity of the grape cane extracts at pH 7.00, and 13.00 opportunistic bacteria, human viruses, and pathogenic yeast were used. The antibacterial tests were performed on Gram-negative and Gram-positive strains, *E. coli* and *S. aureus*, respectively (Figure S1). These bacterial strains have been selected because they represent the main bacteria representative of the two Gram categories. Extracts were tested at different concentrations, starting from 1 to 100 µg/mL. In all the tested conditions, a moderate effect was only observed with “Aglianico” extract at pH 7.00 against *S. aureus*.

When 100 µg/mL was used, the bacterial growth decreased around 40% compared to the respective positive control (only bacterium), while in the other conditions, no significant variation was obtained compared to the controls (Figure S1B).

Indeed, when 100 µg/mL was used, the bacterial growth decreased by around 40% compared to the respective positive control (only bacterium), while in the other conditions, no significant variation was obtained compared to the controls (Figure S1). This moderate reduction in the bacterial growth, which, however, remains below the 50% activity threshold line, could be explained by the nature of the bacterial wall of *S. aureus*, consisting only of a peptidiglycone layer and lacking the outermost lipopolysaccharide (LPS) layer, typical of Gram-negative bacteria. Moreover, no significant results against *Candida albicans* (*C. albicans*) were observed with the extracts at any concentration (Figure S2). Data were statistically significant with *p* ≤ 0.05. The effect against bacteria and fungi were previously described, attesting antimicrobial activity towards these microorganisms, even if the extract concentrations tested were high in comparison to those of the present work. Grape cane extracts prepared with different techniques (microwave-assisted extraction, MAE; conventional extraction, CE; and subcritical water extraction, SWE) showed antibacterial activity with minimal inhibitory concentration (MIC) ranging from 0.73 (*E. coli* ESA 37 cephalosporins-resistant-MAE) to 10.9 mg/mL (*S. mitis* ESA65-SWE). The extracts were also active against *C. albicans* but at high extract concentrations comprised between 2.42 (*C. albicans* ATCC 10231-MAE) and 16.8 mg/mL (Amphotericin B-resistant *C. albicans* ESA 100-SWE) [16]. The antibacterial activity of grape canes from a Spanish *Vitis vinifera* variety, extracted with ethyl acetate, was attested by Gullón et al. [42]. The MIC varied from 5 (*S. aureus* and *Listeria innocua*) to 15 mg/mL (*E. coli*).

**Figure 6:**
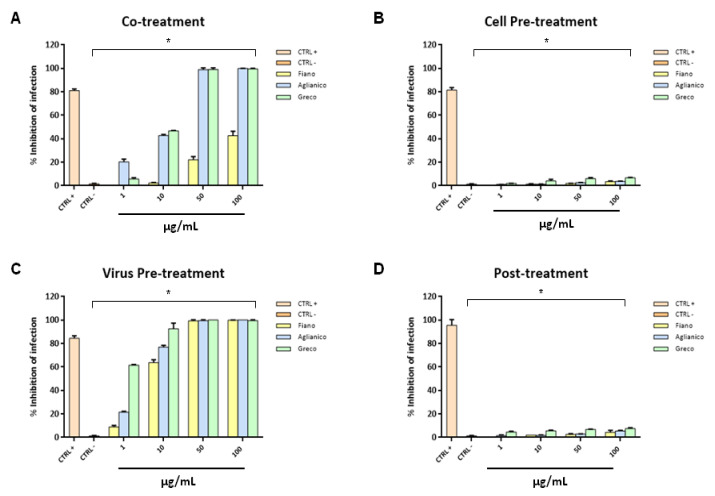
Antiviral activity against HSV-1 of extracts at pH 7.00. Different assays were performed in order to evaluate anti-HSV-1 activity. Extracts inhibited the early stages of infection, acting in co-treatment (**A**) and virus pre-treatment (**C**) assays. Extracts were not able to interact with the cellular surface (**B**) or block the viral replication (**D**). Statistical differences were evaluated via two-way ANOVA; a value of *p* ≤ 0.05 was considered significant, with * *p* ≤ 0.0001.

The antiviral effect of the grape cane extracts was evaluated against HSV-1 and HSV-2. The ability of interfering with HSV-1’s life-cycle through a co-treatment assay was preliminary investigated for extracts at pH 7.00 (Figure 6A). Virus and extracts at the indicated concentrations were incubated together on the cells for 1 h at 37 °C. Setting 50% of viral inhibition as the threshold line, “Fiano” extract was the least effective against HSV-1, while the other two grape cane extracts showed an effective dose-dependent inhibition in HSV-1 replication. Their inhibitory activity was very similar: both “Aglianico” and “Greco” extracts exhibited a half-maximal inhibitory concentration (IC50) at 10 µg/mL, and they were able to totally inhibit HSV-1 infection at the higher concentrations.

To evaluate the mechanism of action of these extracts more profoundly, further experiments were carried out. A cell pre-treatment test was performed to investigate whether the extracts more active in the co-treatment assay could interact with the cell mechanisms inhibiting the viral infection (Figure 6B). This technical approach focuses on the possibility that compounds contained in the extracts and their mixtures are able to interact with the cell surface without penetrating. In this assay, cell monolayers were firstly precooled, and then incubated with the extracts at pH 7.00 for 1 h at 4 °C, and, subsequently, they were infected with viruses at 37 °C. The incubation at 4 °C is essential to allow extracts interaction with the cell wall, but not their entry into the host cell, which, on the contrary, can happen together with the viral infection. Results indicated that no extract had an inhibitory effect on cell infection. Based on this evidence, we can assert that no cell surface mechanism is involved in the HSV-1 infection inhibition due to the grape cane extract treatment. 

The probability that HSV-1 inhibition could be addressed to an interaction among the extract itself and the virus surface was subsequently investigated. In order to verify this possible mechanism of action, a virus pre-treatment assay was performed (Figure 6C). In this approach, the virus was incubated with the grape cane extracts at pH 7.00 for 1 h at 37 °C; then, the mixture was diluted and added onto the Vero cellular monolayer. All extracts showed a strong virucidal activity against HSV-1; in fact, all extracts were able to modulate the HSV-1 infection, including “Fiano” which was not active in the co-treatment assay. It exhibited high antiviral activity with 100% inhibition of virus plaques at 50 μg/mL. In detail, among the three tested extracts, “Greco” was the most active to interfere directly with the viral particles, as it showed 100% inhibition of virus plaques at the lower concentration of 10 μg/mL.

Finally, we investigated the ability of extracts at pH 7.00 to affect HSV-1 replication through a post-treatment assay (Figure 6D). In this assay, the extracts were added to the cells after viral infection, but they did not inhibit its replication and cell-to-cell spread.

**Figure 7:**
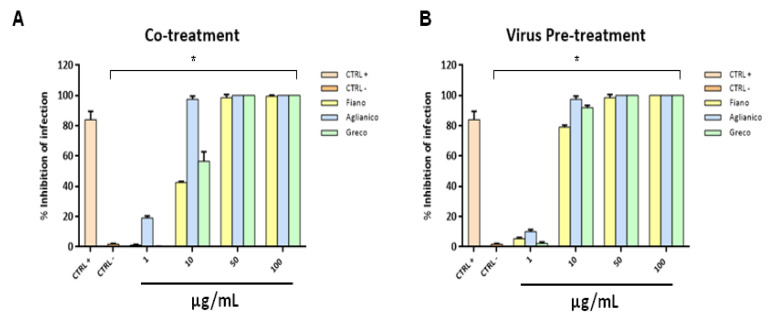
Antiviral activity against HSV-2 of extracts at pH 7.00. Co-treatment (**A**) and virus pre-treatment (**B**) assays were performed. Extracts inhibited the early stages of infection, directly blocking the viral particles. Statistical differences were evaluated via two-way ANOVA; a value of *p* ≤ 0.05 was considered significant, with * *p* ≤ 0.0001.

The antiviral potential of “Aglianico”, “Fiano” and “Greco” extracts at pH 7.00 against HSV-2, which represents the major causative agent of genital herpes, was also investigated. In the co-treatment assay (Figure 7A), “Aglianico” was the most active by completely blocking HSV-2 infection at 10 µg/mL, and “Fiano” and “Greco” were able to halve the viral replication at the same concentration. All the examined extracts showed 100% inhibition at higher concentrations. Virus pre-treatment also represents the election test with HSV-2, indicating that these natural compounds have a direct effect on the viral particle by considerably reducing viral replication at 10 μg/mL (Figure 7B).

**Figure 8:**
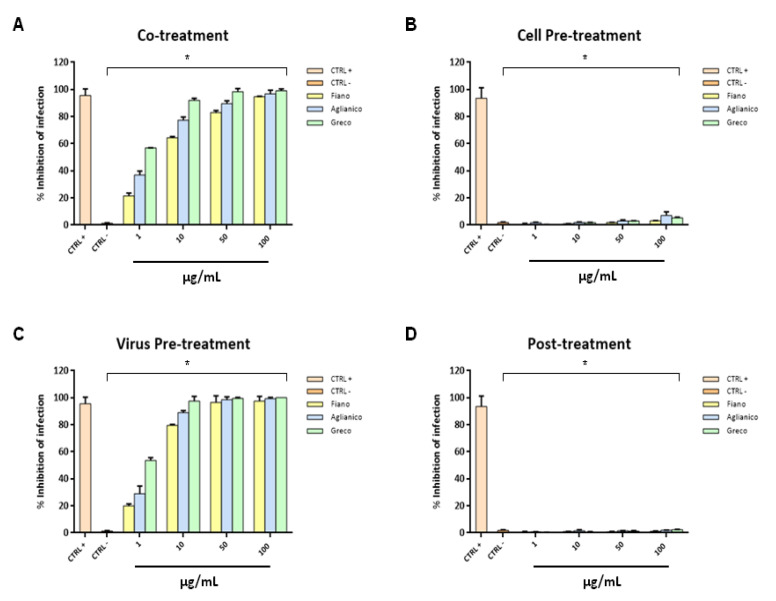
Antiviral activity against HSV-1 of extracts at pH 13.00. Different assays were performed in order to evaluate anti-HSV-1 activity. Extracts inhibited the early stages of infection, acting in co-treatment (**A**) and virus pre-treatment (**C**) assays. Extracts were not able to interact with the cellular surface (**B**) or block the viral replication (**D**). Statistical differences were evaluated via two-way ANOVA; a value of *p* ≤ 0.05 was considered significant, with * *p* ≤ 0.0001.

The antiviral activity against HSV-1 was also evaluated by extracts at pH 13.00 through the above-described assays. In contrast to what was observed at pH 7.00, “Fiano” showed a significant antiviral effect at 10 µg/mL in co-treatment assay, indicating that some components present in this extract are enriched at basic pH. “Aglianico” and “Greco” extracts also showed higher activity compared to the findings obtained at pH 7.00 by almost totally blocking HSV-1 infection at 10 µg/mL (Figure 8A). The response of virus pre-treatment assay was very similar to that at pH 7.00: all three grape cane extracts were able to act directly with HSV-1 particles by blocking the viral replication at 10 µg/mL (Figure 8C). “Greco” extract was the most active by showing an IC50 at 0.9 µg/mL. Extracts at pH 13.00, as those at pH 7.00, were not able to interact with the cell surface in cell pre-treatment or block the viral replication phase in post-treatment assays (Figure 8B,D). Data were statistically significant with *p* ≤ 0.05.

Our findings showed that the grape cane extracts may act either on virus attachment or directly on virus particle as a virucide, inactivating it irreversibly (virus pre-treatment), excluding that they could stably interact with one or more cellular component (cell pre-treatment) or to affect HSV replication (post-treatment). Furthermore, the extracts exhibited a slightly different response against HSV-1 and HSV-2, in agreement with Leary et al. [43], who reported that antiviral compounds could act in a different manner, although these viruses are highly similar both structurally and genetically.

Several authors investigated the antibacterial and antifungal activity of grape cane extracts, but no data about the antiviral effect have been described until now. The first evidence about viral inhibition was reported by Konowalchuk and Speirs [44] in grape samples (extract and juice) and red wines. These samples showed antiviral activity against poliovirus and HSV-1. The filtration of the tested samples on ultrafiltration membranes reduced the activity, indicating that molecules with high molecular weight could be responsible for the observed effect. In addition, the authors supposed that the activity could be related to the protein-binding capacity of some phenolic compounds. We hypothesize that the strong effect observed with the grape cane extracts could be due to the presence of condensed tannins (proanthocyanidins), well-known for their ability to bind proteins. Furthermore, the slightly enhanced effect measured with extracts at pH 13.00 may be due to the favoured extraction at this pH of the stilbenoid resveratrol and its derivatives as the antiviral activity of these compounds has already been described. The antiviral effect of resveratrol tetramers against hepatitis C virus (HCV) was ascertained in studies by Lee et al. [45]. It was shown that the activity was due to the capacity of the resveratrol derivative to bind the HCV helicase, thus blocking virus replication. The inhibition of herpes virus replication by stilbenoid oligomers was proved by Chen et al. [46]. A series of oligomers of resveratrol were tested against HSV-1 and HSV-2, and trimers and tetramers were effective against the virus replication. In a recent review by Annunziata et al. [47], an overview about the antiviral effect of resveratrol against herpes simplex virus is reported. The virus replication in infected Vero cells was highly reduced when 25 and 50 μg/mL of resveratrol were used. In vivo studies on mice demonstrated the effectiveness of resveratrol against HSV infection. Trials carried out by treating HSV lesions on mice with a cream containing resveratrol up to 25% produced a reduction of the injury for both skin (HSV-1) and vaginal (HSV-2) lesions.

## 3. Materials and Methods 

### 3.1. Chemicals

Chemicals needed for phenolic families determination (Folin–Ciocalteu reagent and Na_2_CO_3_ for phenolic compounds; NaNO_2_, AlCl_3_·6H_2_O, and NaOH for flavonoids; HCl, Na_2_MoO_4_ and caffeic acid for *ortho*-diphenols; cinchonine hemisulfate and formaldehyde for tannins), 2,2-diphenyl-1-picrylhydrazyl (DPPH∙), 2,4,6-tripyridyl-S-triazine (TPTZ), FeCl_3_·6H_2_O, ascorbic acid, butylatedhydroxytoluene (BHT), HPLC standards (gallic acid, protocatechuic acid, ellagic acid, *p*-coumaric acid, syringic acid, caffeic acid, quercetin, catechin and resveratrol), brain heart infusion (BHI), agar, glucose, 3-(N-morpholino)propanesulfonic acid (MOPS), and dimethyl sulfoxide (DMSO) were purchased from Sigma-Aldrich Co. (Milan, Italy). High-performance liquid chromatography (HPLC)-grade acetonitrile was obtained from Merck (Darmstadt, Germany). Glacial acetic acid was purchased from Carlo Erba (Rodano, Milan, Italy). HPLC grade water (18.2 MΩ) was prepared by using a Millipore Milli-Q purification system (Millipore Corp., Bedford, MA, USA). All materials used for cell culture and antifungal assay, phosphate-buffered saline (PBS), trypsin-EDTA, Dulbecco’s modified Eagle medium (DMEM), Roswell Park Memorial Institute (RPMI) 1640, and fetal bovine serum (FBS) were purchased from HIMEDIA (Mumbai, India). The β-Gal assay kit and MTT were acquired from ThermoFisher (Waltham, MA, USA).

### 3.2. Extraction of Phenolic Compounds 

Grape canes used in the present study were kindly provided by a winery located in the Campania region (Atripalda, Avellino, Southern Italy). They were collected in February 2017 from the following cultivars: “Aglianico”, “Fiano”, and “Greco”. The material was cut into pieces of 0.5 to 1.0 cm and dried in an oven at 55 °C until reaching constant weight. Then, the samples were powdered with a MF10 IKA mill (Sigma-Aldrich Co. Milan, Italy), sieved to screen particles until 500 μm in size, and used for the extraction of bioactives at different pH (1.00–13.00). Powdered grape canes (2.5 g) were suspended in 20 mM of the following buffers (50 mL): KCl/HCl pH 1.00, citrate/phosphate pH 3.00, 5.00, 7.00, Tris/HCl pH 9.00, NaHCO_3_/NaOH pH 11.00, KCl/NaOH pH 13.00. The suspensions were heated at 50 °C for 20 min under continuous stirring; then, they were cooled on ice and centrifuged at 18,000 rpm for 1 h at 4 °C (Sorvall RC6 plus). The supernatants, after correction of the pH to 7.00, were lyophilized in an Edwards Modulyo freeze-dryer (Edwards, Cinisello Balsamo, Milano, Italy), and the dry extracts (DEs) were stored at 4 °C until use.

### 3.3. Characterization of the Extracts

For grape cane extract characterization, DEs were dissolved in deionized water at a concentration of 10 mg/mL. All tests were conducted in triplicate and the results were expressed as mean ± standard deviation (SD).

#### 3.3.1. Total Phenolic Content 

The phenolic content was measured by the Folin–Ciocalteu method [48]. Aliquots of extract, diluted to 150 μL with deionized water, were mixed with 750 μL of Folin–Ciocalteu reagent (diluted ten-fold with deionized water) and 600 μL of 7.5% (*w*/*v*) Na_2_CO_3_. The reaction was developed at 25 °C for 2 h in the dark, and the absorbance was read at 765 nm against a blank prepared with 150 mL of deionized water (Thermo Scientific spectrophotometer, model Genesys 180, Rodano, Milan, Italy). The total phenolic content was estimated by a calibration curve built with increasing quantities of a standard solution of gallic acid (range 1.5 to 10 μg) and the results were expressed as mg GAE (Gallic Acid Equivalents)/g DE.

#### 3.3.2. Total Ortho-Diphenolic Content

The *ortho*-diphenolic content was measured as described by Arnow [49]. Briefly, 100 μL of extract were diluted to 400 μL with deionized water. Then, 400 μL of 0.5 M HCl (solution A), 400 μL of 1.45 M NaNO_2_ and 0.4 M Na_2_MoO_4_ (solution B), and 400 μL of 1 M NaOH (solution C) were added in sequence. The resulting mixture was immediately read at 500 nm using a blank containing 400 μL of deionized water. The quantification was carried out by a calibration curve obtained with increasing quantities of a standard solution of caffeic acid (range 5–50 μg). The results were expressed as mg CAE (Caffeic Acid Equivalents)/g DE, and subsequently converted, through a conversion factor, to mg GAE in order to compare the amount of *ortho*-diphenols with the total phenolic content. One milligram of CAE corresponded to 0.86 mg GAE.

#### 3.3.3. Total Flavonoid Content 

The flavonoid content was determined following the method of Barreira et al. [50] with some modifications. Briefly, 250 μL of extract were mixed with 1.25 mL of deionized water and 75 μL of 5% (*w*/*v*) NaNO_2_. After 5 min, 150 μL of 16% (*w*/*v*) AlCl_3_ were added. After 1 min, 500 μL of 1 M NaOH and 275 μL of deionized water were added, and the resulting solution was vigorously mixed. The absorbance was read at 510 nm versus a blank containing 250 μL of deionized water. The flavonoid amount was determined by a calibration curve obtained with increasing quantities of a standard solution of catechin (range 5 to 75 μg). The results were expressed as mg CE (Catechin Equivalents)/g DE and subsequently converted, through a conversion factor, to mg GAE in order to compare the amount of flavonoids with the total phenolic content. One milligram of CE corresponded to 0.93 mg GAE.

#### 3.3.4. Total Tannin Content

The total tannin content was estimated according to Peri and Pompei [51], with some modifications. Briefly, 0.8 mL of phenolic extract were added to 0.8 mL of 0.5% (*w/v*) cinchonine hemisulfate in a 2 mL Eppendorf tube. The solution was mixed and left overnight at 4 °C in order to achieve a better precipitation. After centrifugation at 13,200 rpm for 5 min at 4 °C, a supernatant containing the non-tannin fraction, and a pellet representing the tannin fraction were obtained. The Folin–Ciocalteu assay was carried out on the supernatant in order to calculate the non-tannin content. The total tannin fraction was determined by the difference between the total phenolic content and the non-tannin content, and results were expressed as mg GAE/g DE. 

#### 3.3.5. Hydrolysable and Condensed Tannin Content 

Hydrolysable and condensed tannin fractions were determined applying the procedure of Peri and Pompei [51] followed by the formaldehyde precipitation method described by Scalbert et al. [52], with some modifications. The tannin residue, obtained after precipitation with the cinchonine hemisulfate, was dissolved in the original sample volume (0.8 mL) with ethanol/water (1:1 *v*/*v*). Then, 0.5 mL of this solution were mixed with 0.25 mL of 37% HCl/water (2:5 *v*/*v*) and 0.25 mL of 4.8% formaldehyde. The resulting mixture was vigorously mixed, incubated overnight at room temperature, and centrifuged at 13,200 rpm, for 30 min at 4 °C. The supernatant (150 μL) was assayed by the Folin–Ciocalteu method, and the value obtained represented the hydrolysable tannin fraction. The condensed tannin content was determined by the difference between the total tannin content and the hydrolysable tannin content. Results were expressed as mg GAE/g DE.

#### 3.3.6. Reversed-Phase High-Performance Liquid Chromatography-Ultraviolet (RP-HPLC–UV) and HPLC-Electrospray Ionization Multistage Ion Trap Mass Spectrometry (HPLC-ESI-ITMSn) Analyses 

Selected grape cane extracts (pH 1.00, pH 7.00, and pH 13.00) from “Aglianico”, “Fiano” and “Greco” cultivars were reconstituted in deionized water and analysed by HPLC/ESI-ITMSn on a Surveyor MS micro HPLC coupled with a LCQ DECA XP Max ion trap mass spectrometer, equipped with Xcalibur^®^ system manager data acquisition software (Thermo Finnigan, San Jose, CA, USA). Individual compounds were separated on a Luna C18 (2) column (250 mm × 4.6 mm, 5.0 μm, Phenomenex, Torrance, CA, USA) equipped with a SecurityGuard™ pre-column containing a C18 cartridge, at a flow rate of 700 μL/min; solvent A was 0.5% acetic acid, and solvent B was 0.1% acetic acid-acetonitrile (1:1 *v*/*v*). After a 5 min hold at 5% solvent B, elution was performed by a linear gradient from 5 to 55% solvent B in 55 min, and from 55 to 95% solvent B in 10 min, followed by 10 min of maintenance. The column effluent was split into two by means of a “T junction” placed after the chromatographic column and analysed “on-line” both by UV and ESI/MS; 80% of the effluent was sent to the UV detector (detection 280 nm) while 20% of the effluent was analysed by ESI/MS. Mass spectra were recorded from a mass-to-charge ratio (*m*/*z*) of 120 to 2000 in negative ionization mode. The capillary voltage was set at −20 V, the spray voltage was 3.5 kV, and the tube lens offset was −10 V. The capillary temperature was 275 °C. Data were acquired in MS, MS/MS, and MSn scanning modes.

#### 3.3.7. Quantification by RP–HPLC–DAD

For quantification of the identified compounds, the HPLC system Dionex UltiMate^®^ 3000, equipped with a quaternary pump and an UltiMate^®^ diode array detector (Dionex, California, USA) was used. The HPLC separation column and the elution program are described in the previous paragraph 3.3.6. The dry extracts were dissolved in deionized water (10 mg/mL), filtered through a Chromafil syringe filter, pore-size 0.45 μm (Macherey-Nagel GmbH & Co., Duren, Germany), and 50 µL were loaded onto the column. For the identified phenolic compounds for which a commercial standard was not available, the quantification was performed through the calibration curve of the most similar available standards. Results were expressed as μg of phenolic compound/g DE.

### 3.4. Antioxidant Activity 

#### 3.4.1. Free-Radical Scavenging Capacity 

The free-radical scavenging activity was evaluated by the 2,2-diphenyl-1-picrylhydrazyl (DPPH∙) assay according to Barreira et al. [50], with some modifications. A volume of extract containing 5 μg GAE of phenolic compounds was opportunely diluted to 150 μL with deionized water and mixed with 1.35 mL of 60 μM DPPH in methanol. The antioxidant activity was evaluated by reading the absorbance at 517 nm for 30 min versus a control containing 150 μL of deionized water. The RSA was calculated according to the following formula:

(1) $$\mathrm{RSA}\;\left(\%\right)=\left(1-\frac{{\mathrm{Absorbance}}_{\mathrm{sample}}}{{\mathrm{Absorbance}}_{\mathrm{control}}}\right)\times 100$$

and compared with the values obtained with 5 μg GAE of the following antioxidants chosen as standards and tested under the same conditions: Q and BHT.

#### 3.4.2. Ferric Reducing Antioxidant Power 

FRAP assay was performed according to Fernández-Agulló et al. [53]. Briefly, the assay solution, containing 300 mM sodium acetate buffer, pH 3.6 (A), 10 mM 2,4,6-tripyridyl-S-triazine (TPTZ) in 40 mM HCl (B), and 20 mM FeCl_3_·6H_2_O (C), was mixed in 10:1:1 (A:B:C) ratio at the time of use. Samples containing 1 mg of solubilized DEs were diluted to 50 μL and added to 1.5 mL of the FRAP solution. After 4 min incubation, the absorbance was read at 593 nm against a blank made of FRAP solution. FRAP was calculated using a calibration curve built with amounts of ascorbic acid ranging from 0.5 to 6 μg, and compared with FRAP values obtained with 1 mg of the standards Q and BHT. The results were expressed as μg AAE/mg DE for extracts and μg AAE/mg for standards.

### 3.5. Antimicrobial Activity Analysis

#### 3.5.1. Bacterial and Fungal Growth 

The bacterial and fungal strains used were *E. coli* ATCC 11219, *S. aureus* ATCC 6538, and *C. albicans* ATCC 90028. To standardize the bacterial cell suspension, the fresh colonies of each strain, grown on BHI Agar plates, were inoculated into BHI broth and cultured overnight at 37 °C in a shaking incubator (Memmert, ICO105MED). Subsequently, the bacterial inoculum was resuspended in the fresh medium and further incubated at 37 °C until reaching the exponential growth phase (6 × 10^8^ CFU/mL). Serial dilutions and subsequent plating were performed in order to determine the final concentration of bacteria (1 × 10^6^ CFU/mL).

For the antifungal activity assay, colonies of *C. albicans* were inoculated in 3 mL of sterile saline, and its turbidity was adjusted to 0.5 McFarland standards (5 × 10^6^ CFU/mL). Serial dilutions, conducted in RPMI 1640 added to 2% glucose and MOPS (0.165 mol/L), were performed in order to determine the final concentration of strains (1 × 10^5^ CFU/mL).

#### 3.5.2. Antibacterial Activity Assay 

Susceptibility testing was performed using the broth micro-dilution method outlined by the National Committee on Clinical Laboratory Standards (NCCLS) using sterile 96-well microliter plates. The dilutions of each extract (in a range of concentration from 1 to 100 μg/mL) were prepared in BHI broth at a volume of 100 μL/well. Each well was inoculated with 50 μL of the standardized bacterial suspension, corresponding to a final test concentration of about 5 × 10^5^ CFU/mL. The antibacterial activity was expressed as the relative percentage (%) of bacterial growth after 20 h of incubation at 37 °C [54,55]. All experiments were performed in triplicate, and results were expressed as mean ± standard deviation (SD).

#### 3.5.3. Antifungal Activity Assay 

Antifungal susceptibility assay was performed using EUCAST guidelines in sterile 96-well microliter plates. The dilutions of each extract (in a range of concentration from 1 to 100 μg/mL) were prepared in RPMI 1640 at a volume of 200 μL/well. Each well was inoculated with 100 μL of the standardized fungal inoculum, corresponding to a final test concentration of about 1 × 10^5^ CFU/mL. The antifungal activity was expressed as the relative percentage (%) of growth observed after 48 h of incubation at 37 °C. All experiments were performed in triplicate, and the results were expressed as mean ± standard deviation (SD).

#### 3.5.4. Cell Culture and Viruses 

Vero cells (epithelial kidney cells of *Cercopithecus aethiops*) supplied by ATCC (CCL-81) were grown in DMEM supplemented with 10% FBS. HSV-1 (strain SC16), and HSV-2 (strain 333), both containing a lac Z gene under the control of the CMV IE-1 promoter for the expression of β-galactosidase, were propagated on Vero monolayers [56]. 

##### Cytotoxicity Assay 

MTT assay was performed to evaluate the cytotoxicity of the extracts. Vero cells were plated in a 96-well flat-bottomed plate (10^4^ cells/well) and incubated overnight at 37 °C/5% CO_2_. Then, the extracts, positive (only medium) and negative (DMSO) controls were added to the wells. In detail, the same increasing concentrations were tested for the different samples (1, 10, 50, 100, 250, 500 and 1000 µg/mL). After 24 h, the MTT (blue) was added for 3 h and cells converted it into Formazan (purple). Finally, DMSO was used to re-solubilize the formazan and the viability was assessed at 570 nm through a Bio-Rad microplate reader (Bio-Rad Laboratories, Hercules, CA, USA). All assays were performed in triplicate and the results were obtained in three independent experiments. The data were reported as a percentage of cell viability compared to the positive control.

##### Virus Entry Assays 

For the following assays, the grape cane extracts were dissolved in DMEM without FBS at the following concentrations: 1, 10, 50 and 100 µg/mL. All experiments were performed in triplicate. The percentage of infectivity inhibition was calculated by counting the number of plaques obtained in the presence of the sample with respect to those in virus control (only virus, negative control). The peptide gH493-512 was used as a positive control in co-treatment and virus pre-treatment assays, while dextran sulphate and acyclovir were used for cell-treatment and post-treatment assay, respectively [57]. In order to evaluate the antiviral activity of the extracts on HSV, four different assays were performed [56,58].

(a) Co-treatment assay. A preliminary assay to assess if the compound has antiviral activity. Vero cells were seeded (3 × 10^5^ cells/well) in 12-well plates and incubated overnight at 37 °C/5% CO_2_. The day after, the extracts and HSV-1 or HSV-2 were added simultaneously to the cell monolayer at multiplicity of infection (MOI) of 0.01 for 1 h at 37 °C.
(b) Virus pre-treatment assay. The assay allows us to evaluate if the extract affects HSV infectivity acting directly on the viral particles. The extracts were incubated in the presence of HSV-1 or HSV-2 (MOI 0.1) at 37 °C for 1 h. Then, the mixtures were diluted with medium and were titrated on Vero cell monolayers.
(c) Cell pre-treatment assay. To assess if the extracts could act on the target cells, pre-chilled Vero cells were incubated with the samples for 1 h at 4 °C. Subsequently, they were removed and the cells were infected with HSV-1 or HSV-2 (MOI 0.01) for 1 h at 37 °C.
(d) Post-treatment assay. The assay allows us to assess if the extract acts on HSV replication. For this purpose, Vero cell monolayers were firstly incubated with HSV-1 or HSV-2 (MOI 0.01) for 1 h at 37 °C. Then, the extracts were added for an additional incubation period of 1 h at 37 °C.

For all above treatments, non-penetrated viruses were inactivated by citrate buffer, pH 3.0 after the incubation time of 1 h at 37 °C with cells. The infected cells were washed with PBS, covered with fresh culture medium supplemented with 5% carboxymethyl cellulose (in 1:2 ratio), and incubated for 24 h. After, monolayers were fixed, stained with X-gal (5-bromo-4-chloro-3-indolyl-β-D-galactopyranoside), and plaques were microscopically scored.

### 3.6. Statistical Analysis

All tests were performed in triplicate and expressed as mean ± standard deviation (SD) calculated by Microsoft Excel. Experimental data were analysed using GraphPad Prism (version 5). Significant differences were determined by two-way analysis of variance (ANOVA) completed by Bonferroni post-tests. Mean values were considered significantly different at *p* ≤ 0.05.

## 4. Conclusions 

In the present study, the valorisation of grape canes, a by-product of vine processing produced in large amounts, was achieved through the production of active aqueous extracts. The importance and dimension of the wine industry worldwide justify the need to redirect this waste, currently not valued, to more significant and environmentally friendly uses. Grape canes from “Aglianico”, “Fiano” and “Greco” cultivars were used to produce extracts with the dual purpose of providing the phenolic profile of each cultivar and testing the extracts as potential antioxidant and antimicrobial agents. The method of extraction here applied is simple, cost-effective and easily scalable. This could make it spread quickly and allow grape canes to have a more noble use. The extracts produced could be safely used as antioxidants in several fields, such as food and cosmetic industries, thus representing a response to the growing attention of consumers to health and the environment, and to the increasing trend of preferring natural preparations to achieve health benefits. Moreover, the remarkable activity registered towards HSV-1 and HSV-2 may facilitate and guide further studies to develop new antimicrobial compounds from agro-industrial residues currently underutilized.

## Figures and Tables

**Table 1:** List of phenolic compounds identified by HPLC-UV and HPLC-ITMS^n^ in “Aglianico” (A), “Fiano” (F), and “Greco” (G) grape canes extracted at pH 1.00, 7.00, and 13.00. Specific quasi-molecular ions and fragment ions are reported for each compound.

Peak	RT	[M-H]^−^ *m*/*z*	MS^n^ Ions *m*/*z*	Identified Compound	pH 1.00			pH 7.00			pH 13.00		
					A	F	G	A	F	G	A	F	G
1	3.98	149	MS^2^ [149]: 87	Tartaric acid	+	+	+	+	+	+	+	+	+
2	5.26	133	MS^2^ [133]: 115	Malic acid	+	+	+	-	+	+	+	+	+
3	5.94	337	MS^2^ [337]: 191, 173, 163	Coumaroylquinic acid	+	+	-	+	+	+	+	-	-
4	7.43	191	MS^2^ [191]: 173	Quinic acid	+	+	+	+	+	+	+	-	+
5	14.48	331	MS^2^ [331]: 169	Galloyl glucose isomer	+	-	+	-	-	+	-	-	-
6	14.99	169	MS^2^ [169]: 125	Gallic acid	-	+	+	+	+	+	-	-	+
7	15.03	865	MS^2^ [865]: 695, 577, 575, 407, 543, 739, 713, 449, 587, 289	Procyanidin trimer B-type isomer	+	-	-	-	-	-	-	-	-
8	18.05	435	MS^2^ [435]: 273	Afzelechin hexoside	+	+	+	+	+	+	+	+	+
9	18.48	331	MS^2^ [331]: 169	Galloyl glucose isomer	+	+	+	+	+	+	+	-	+
10	20.43	865	MS^2^ [865]: 695, 577, 575, 407, 543, 739, 713, 449, 587, 289	Procyanidin trimer B-type isomer	+	+	+	+	+	+	+	+	-
11	21.42	315	MS^2^ [315]: 153	Protocatechuic acid glucoside	+	+	+	+	+	+	+	+	+
12	21.59	865	MS^2^ [865]: 695, 577, 575, 407, 543, 739, 713, 449, 587, 289	Procyanidin trimer B-type isomer	-	-	+	-	-	-	-	-	-
13	21.64	575	MS^2^ [575]: 449, 407, 289, 287, 285	Procyanidin dimer A-type isomer	-	-	-	-	+	+	-	-	-
14	23.51	331	MS^2^ [331]: 169	Galloyl glucose isomer	+	+	+	+	+	-	+	-	+
15	23.52	879	MS^2^ [879]: 727, 709, 547	Procyanidin dimer digallate A-type isomer	-	-	+	-	-	-	-	-	-
16	24.11	593	MS^2^ [593]: 575, 467, 423, 305	(Epi)catechin-(epi)gallocatechin	-	-	-	+	-	-	-	-	-
17	24.50	315	MS^2^ [315]: 153	Protocatechuic acid glucoside	+	-	-	+	-	-	+	-	-
18	24.73	879	MS^2^ [879]: 727, 709, 547	Procyanidin dimer digallate A-type isomer	-	-	+	-	-	+	-	-	-
19	25.44	879	MS^2^ [879]: 727, 709, 547	Procyanidin dimer digallate A-type isomer	-	-	-	-	-	+	-	-	-
20	25.57	359	MS^2^ [359]: 197	Syringic acid hexoside	+	+	+	+	+	+	+	+	+
21	26.12	593	MS^2^ [593]: 575, 467, 423, 305	(Epi)catechin-(epi)gallocatechin	-	-	-	+	-	-	-	-	-
22	27.98	311	MS^2^ [311]: 179, 149	Trans-caffeoyltartaric acid (caftaric acid)	+	+	+	+	+	+	+	+	+
23	28.90	593	MS^2^ [593]: 575, 467, 423, 305	(Epi)catechin-(epi)gallocatechin	+	-	+	+	-	+	+	-	+
24	30.27	1153	MS^2^ [1153]: 1027, 1001, 983, 865, 863, 577, 575	Procyanidin tetramer B-type isomer	-	-	-	+	+	-	-	-	-
25	30.56	1153	MS^2^ [1153]: 1027, 1001, 983, 865, 863, 577, 575	Procyanidin tetramer B-type isomer	-	-	-	+	-	+	-	-	-
26	30.62	577	MS^2^ [577]: 425, 407, 289	Procyanidin dimer B-type isomer	+	+	+	+	+	+	-	-	-
27	31.37	613	MS^2^ [613]: 451, 289	(Epi)catechin-3-*O*-dihexoside	+	-	+	-	-	-	-	-	-
28	32.37	577 465	MS^2^ [577]: 425, 407, 289 MS^2^ [465]: 303	Procyanidin dimer B-type isomer Dihydroquercetin hexoside	+ +	- -	+ +	+ +	+ -	+ -	- +	- -	- +
29	32.72	865	MS^2^ [865]: 695, 577, 575, 407, 543, 739, 713, 449, 587, 289	Procyanidin trimer B-type isomer	-	-	-	+	+	+	-	-	-
30	33.85	325	MS^2^ [325]: 163	*p*-Coumaric acid glucoside	-	-	+	-	-	-	-	-	-
31	34.54	289	MS^2^ [289]: 245, 205, 179, 125	Catechin	+	+	+	+	+	+	+	+	+
32	34.92	1153	MS^2^ [1153]: 1027, 1001, 983, 865, 863, 577, 575	Procyanidin tetramer B-type isomer	-	-	-	+	+	-	-	-	-
33	35.08	865	MS^2^ [865]: 695, 577, 575, 407, 543, 739, 713, 449, 587, 289	Procyanidin trimer B-type isomer	+	-	+	+	+	+	-	-	-
34	35.20	295	MS^2^ [295]: 163	*trans*-Coumaroyltartaric acid (coutaric acid)	+	-	-	-	-	-	-	-	-
35	36.07	1153	MS^2^ [1153]: 1027, 1001, 983, 865, 863, 577, 575	Procyanidin tetramer B-type isomer	-	-	-	-	-	+	-	-	-
36	35.99	577	MS^2^ [577]: 425, 407, 289	Procyanidin dimer B-type isomer	-	-	-	+	-	+	-	-	-
37	36.64	879	MS^2^ [879]: 727, 709, 547	Procyanidin dimer digallate A-type isomer	-	-	-	-	+	+	-	-	-
38	37.76	577	MS^2^ [577]: 425, 407, 289	Procyanidin B2	+	+	+	+	+	+	+	-	-
39	38.03	449	MS^2^ [449]: 287, 269, 259	Dihydrokaempferol hexoside or Eriodictyol hexoside	+	-	-	+	-	-	+	-	-
40	38.19	1153	MS^2^ [1153]: 1027, 1001, 983, 865, 863, 577, 575	Procyanidin tetramer B-type isomer	-	-	-	+	-	-	-	-	-
41	39.26	865	MS^2^ [865]: 695, 577, 575, 407, 543, 739, 713, 449, 587, 289	Procyanidin trimer B-type isomer	-	-	-	-	-	+	-	-	-
42	39.27	521	MS^2^ [521]: 359, 223	Rosmarinic acid hexoside	+	+	+	+	+	-	-	-	-
43	39.68	1153	MS^2^ [1153]: 1027, 1001, 983, 865, 863, 577, 575	Procyanidin tetramer B-type isomer	-	-	-	-	-	+	-	-	-
44	40.66	289	MS^2^ [289]: 245, 205, 179, 125	Epicatechin	+	+	+	+	+	+	+	-	-
45	41.59	389	MS^2^ [389]: 299, 269 MS^3^ [269]: 241, 175, 163	Resveratrol-*C*-glucoside	+	+	+	+	+	+	+	+	+
46	42.14	729	MS^2^ [729]: 603, 577, 559, 451, 441, 425, 407, 289	Procyanidin dimer monogallate B-type isomer	-	-	-	-	-	+	-	-	-
47	42.59	879	MS^2^ [879]: 727, 709, 547	Procyanidin dimer digallate A-type isomer	-	-	-	+	-	+	-	-	-
48	42.68	865	MS^2^ [865]: 695, 577, 575, 407, 543, 739, 713, 449, 587, 289	Procyanidin trimer B-type isomer	+	-	+	+	+	+	-	-	-
49	43.72	1153	MS^2^ [1153]: 1027, 1001, 983, 865, 863, 577, 575	Procyanidin tetramer B-type isomer	-	-	-	+	+	+	-	-	-
50	43.88	729	MS^2^ [729]: 603, 577, 559, 451, 441, 425, 407, 289	Procyanidin dimer monogallate B-type isomer	-	-	-	+	+	+	-	-	-
51	46.38	433	MS^2^ [433]: 301 MS^3^ [301]: 284, 257, 229, 185	Ellagic acid pentoside	-	-	-	+	+	+	+	+	+
52	48.02	575	MS^2^ [575]: 449, 407, 289, 287, 285	Procyanidin dimer A-type isomer	+	+	+	-	-	-	-	-	-
53	49.80	609 419	MS^2^ [609]: 447, 301 MS^2^ [419]: 287, 269	Rutin (quercetin-3-*O*-rutinoside) Dihydrokaempferol-*O*-pentoside or Eriodictyol pentoside	+ +	- +	- +	- -	- -	- -	- -	- -	- -
54	49.82	301	MS^2^ [301]: 257, 229, 185	Ellagic acid	-	-	-	+	-	-	+	+	+
55	50.66	389	MS^2^ [389]: 289, 227	Resveratrol-*O*-glucoside (Piceid)	+	+	-	+	-	-	+	+	+
56	50.98	865	MS^2^ [865]: 695, 577, 575, 407, 543, 739, 713, 449, 587, 289	Procyanidin trimer B-type isomer	-	-	-	-	+	+	-	-	-
57	50.99	449	MS^2^ [449]: 287, 269, 259	Dihydrokaempferol hexoside or Eriodictyol hexoside	+	-	-	+	-	+	-	-	-
58	51.21	575	MS^2^ [575]: 449, 407, 289, 287, 285	Procyanidin dimer A-type isomer	-	-	+	-	-	-	-	-	-
59	51.32	463	MS^2^ [463]: 301 MS^3^ [301]: 179, 151	Isoquercitrin (quercetin-3-*O*-glucoside)	+	+	+	+	+	+	-	-	-
60	52.78	449	MS^2^ [449]: 287, 269, 259	Dihydrokaempferol hexoside or Eriodictyol hexoside	-	-	-	+	-	+	-	-	-
61	53.54	469	MS^2^ [469]: 451, 411, 375	Resveratrol dimer	+	-	-	-	-	-	-	-	-
62	54.46	477	MS^2^ [477]: 301 MS^3^ [301]: 179, 151	Quercetin 3-glucuronide	+	+	-	+	+	+	+	-	-
63	55.80	469	MS^2^ [469]: 451, 363, 375 MS^3^ [451]: 423, 357 MS^4^ [357]: 329, 263 MS^3^ [363]: 345, 269 MS^4^ [345]: 330, 327	Resveratrol dimer (caraphenol)	+	-	+	+	+	+	+	+	+
64	57.89	433	MS^2^ [433]: 271 MS^3^ [271]: 177, 151	Naringenin-*O*-hexoside	+	-	-	+	-	-	+	-	-
65	59.97	575	MS^2^ [575]: 449, 407, 289, 287, 285	Procyanidin dimer A-type isomer	-	+	+	-	-	-	-	-	-
66	61.54	453	MS^2^ [453]: 435, 359	Resveratrol dimer	-	-	-	-	-	-	+	-	-
67	66.48	227	MS^2^ [227]: 185, 183, 159, 157	Resveratrol	+	+	+	+	+	+	+	+	+
68	69.15	923	MS^2^ [923]: 905, 881, 801, 783, 707, 689 MS^3^ [805]: 863, 783 MS^4^ [863]: 821, 741	Viniferol E	+	+	+	+	+	+	+	+	+
69	70.35	905	MS^2^ [905]: 811, 717, 451, 357 MS^3^ [811]: 717 MS^4^ [717]: 675, 611	Resveratrol tetramer	+	-	-	+	+	+	+	+	+
70	72.30	453	MS^2^ [453]: 435, 411, 369, 359, 347, 253	ε-Viniferin	+	+	+	+	+	+	+	+	+
71	72.61	905	MS^2^ [905]: 811, 799, 545, 451, 359 MS^3^ [811]: 793, 717, 705	Resveratrol tetramer	-	-	-	-	-	-	+	-	+
72	72.82	905	MS^2^ [905]: 811, 799, 545, 451, 359 MS^3^ [811]: 793, 717, 705	Resveratrol tetramer	-	-	-	-	-	-	+	-	-
73	72.98	453	MS^2^ [453]: 435, 411, 369, 359, 347, 253	Resveratrol dimer	-	-	-	-	-	-	+	+	+
Number of compounds detected in the individual extracts					42	27	37	48	37	46	33	18	24

+ = detected; - = not detected.

**Table 2:** Quantification of main phenolic compounds identified in “Aglianico”, “Fiano”, and “Greco” grape canes extracted at pH 1.00, 7.00, and 13.00.

Identified Compound (µg Equivalent/g DE)	Aglianico	pH 1.00 Fiano	Greco	Aglianico	pH 7.00 Fiano	Greco	Aglianico	pH 13.00 Fiano	Greco
^a^ Coumaroylquinic acid	145.62 ± 0.77 a	171.23 ± 47.38 d	163.61 ± 8.25 f	397.37 ± 32.23 a	367.28 ± 14.20 d	302.54 ± 60.28 f	290.20 ± 23.48 a	n.d.	185.12 ± 9.65 f
^b^ Galloyl glucose isomer	1210.42 ± 165.80 a	n.d.	1738.51 ± 64.63 f	n.d.	n.d.	54.60 ± 3.96 g	n.d.	n.d.	n.d.
^b^ Gallic acid	n.d.	2143.61 ± 139.10 d	n.m.	4209.78 ± 376.14	4492.14 ± 531.24 e	4057.35 ± 143.12 f	n.d.	n.d.	2460.78 ± 160.31 g
^c^ Procyanidin trimer B-type isomer	n.m.	n.m.	n.m.	n.m.	n.m.	745.82 ± 67.05	n.m.	n.m.	n.d.
^d^ Protocatechuic acid glucoside	n.m.	n.m.	n.m.	n.m.	n.m.	n.m.	630.71 ± 18.50	n.m.	356.12 ± 2.82
^b^ Galloyl glucose isomer	n.m.	n.m.	n.m.	n.m.	407.65 ± 38.38	n.d.	n.m.	n.d.	119.56 ± 26.68
^c^ Procyanidin dimer digallate A-type	n.d.	n.d.	n.m.	n.d.	n.d.	499.38 ± 38.57	n.d.	n.d.	n.d.
^f^ Syringic acid hexoside	n.m.	n.m.	n.m.	n.m.	n.m.	55.12 ± 1.85 f	68.12 ± 11.27	43.29 ± 11.34	82.66 ± 10.52 f
^e^ trans-caffeoyltartaric acid (caftaric acid)	200.17 ± 49.42 a	170.93 ± 5.56 d	136.67 ± 11.22 f	792.32 ± 27.58 b	2465.38 ± 86.23 e	466.41 ± 3.89 f	89.06 ±20.49 a	472.62 ± 33.27 d	144.80 ± 20.31 f
^c^ (epi)catechin- (epi)gallocatechin	203.17 ± 21.44 a	n.d.	152.47 ± 42.30	1806.99 ± 254.51 b	n.d.	n.m.	32.25 ± 2.65 a	n.d.	n.m.
^c^ Procyanidin tetramer B-type isomer	n.d.	n.d.	n.d.	1059.04 ± 217.77	1747.51 ± 362.76	1308.36 ± 63.98	n.d.	n.d.	n.d.
^c^ Procyanidin dimer B-type isomer	786.64 ± 60.58 a	217.05 ± 12.76 d	124.90 ± 7.09 f	2852.34 ± 352.60 b	1133.51 ± 234.94 e	2571.94 ±3 35.22 g	n.d.	n.d.	n.d.
^c^ (Epi)catechin- 3-*O*-dihexoside	375.57 ± 51.74	n.d.	342.74 ± 36.90	n.d.	n.d.	n.d.	n.d.	n.d.	n.d.
^c^ Procyanidin dimer B-type isomer	495.09 ± 20.70 a	n.d.	339.46 ± 15.40 f	3056.73 ± 563.63 b.	1337.50 ± 410.72	2010.59 ± 408.81 g	n.d.	n.d.	n.d.
^g^ Dihydroquercetin hexoside	n.m.	n.d.	53.39 ± 8.16 f	n.m.	n.m.	n.d.	128.87 ± 3.72	n.d.	135.05 ± 14.52 f
^c^ Procyanidin trimer B-type isomer	n.d.	n.d.	n.d.	2226.55 ± 428.60	444.30 ± 114.2	935.60 ± 144.07	n.d.	n.d.	n.d.
^c^ Catechin	1344.16 ± 11.36 a	439.87 ± 71.51 d	n.m.	3354.62 ± 474.37 b	4195.19 ± 475.23 e	4248.00 ± 6.96 f	406.52 ± 90.26 c	151.33 ± 45.66 d	478.19 ± 9.13 g
^c^ Procyanidin tetramer B-type isomer	n.d.	n.d.	n.d.	533.83 ± 22.84	n.d.	n.d.	n.d.	n.d.	n.d.
^c^ Procyanidin trimer B-type isomer	257.00 ± 9.60	n.d.	n.m.	n.m.	1331.38 ± 314.18	3438.21 ± 435.31	n.d.	n.d.	n.d.
^c^ Procyanidin tetramer B-type isomer	n.d.	n.d.	n.d.	n.d.	713.08 ± 160.91	1675.82 ± 624.35	n.d.	n.d.	n.d.
^c^ Procyanidin dimer B-type isomer	n.d.	n.d.	n.d.	610.46 ± 176.26	n.d.	140.14 ± 14.31	n.d.	n.d.	n.d.
^c^ Procyanidin dimer digallate A-type	n.d.	n.d.	n.d.	n.d.	1806.73 ± 338.36	1573.46 ± 292.54	n.d.	n.d.	n.d.
^c^ Procyanidin B2	475.98 ± 105.43 a	n.m.	n.m.	1346.41 ± 4.35 b	1706.31 ± 355.72	2185.02 ± 243.37	516.84 ± 62.75 a	n.d.	n.d.
^g^ Dihydrokaempferol hexoside or Eriodictyol hexoside	16.62 ± 2.54 a	n.d.	n.d.	140.14 ± 14.31 a	n.d.	n.d.	107.62 ± 12.54 a	n.d.	n.d.
^c^ Procyanidin trimer B-type isomer	n.d.	n.d.	n.d.	n.d.	n.d.	1423.23 ± 507.39	n.d.	n.d.	n.d.
^h^ Rosmarinic acid hexoside	276.14 ± 21.12	67.43 ± 13.12 d	314.92 ± 13.80	n.m.	3005.53 ± 128.41 e	n.d.	n.d.	n.d.	n.d.
^c^ Procyanidin tetramer B-type isomer	n.d.	n.d.	n.d.	n.d.	n.d.	1435.38 ± 397.16	n.d.	n.d.	n.d.
^c^ Epicatechin	26.41 ± 5.69 a	534.06 ± 11.51 d	706.44 ± 179.01 f	1127.47 ± 139.79 b	2670.85 ± 546.41 e	5399.49 ± 135.21 g	967.32 ± 37.50 b	n.d.	n.d.
^i^ Resveratrol-*C*-glucoside	207.93 ± 13.27 a	88.75 ± 4.75 d	112.57 ± 19.00 f	464.39 ± 23.42 a	326.22 ± 25.20 d	396.78 ± 21.80 f	147.17 ± 3.50 a	87.72 ± 3.54 d	111.63 ± 9.14 f
^c^ Procyanidin dimer digallate A-type	n.d.	n.d.	n.d.	81.50 ± 2.21	n.d.	n.m.	n.d.	n.d.	n.d.
^c^ Procyanidin trimer B-type isomer	130.74 ± 3.22 a	n.d.	154.66 ± 77.71 f	576.81 ± 113.12 b	641.05 ± 46.07	2205.38 ± 169.12 g	n.d.	n.d.	n.d.
^c^ Procyanidin tetramer B-type isomer	n.d.	n.d.	n.d.	203.92 ± 39.80	336.33 ± 20.87	n.m.	n.d.	n.d.	n.d.
^c^ Procyanidin dimer monogallate	n.d.	n.d.	n.d.	255.99 ± 18.36	379.53 ± 136.64	3151.48 ± 163.54	n.d.	n.d.	n.d.
^j^ Ellagic acid pentoside	n.d.	n.d.	n.d.	174.86 ± 0.66 a	39.64 ± 10.26 d	159.41 ± 2.05 f	354.44 ± 3.80 a	181.97 ± 9.71 d	95.75 ± 18.77 f
^c^ Procyanidin dimer A-type	187.88 ± 3.76	40.87 ± 7.29	152.61 ± 8.44	n.d.	n.d.	n.d.	n.d.	n.d.	n.d.
^k^ Rutin (quercetin-3-*O*-rutinoside)	69.18 ± 5.04	n.d.	n.d.	n.d.	n.d.	n.d.	n.d.	n.d.	n.d.
^g^ Dihydrokaempferol-*O*-pentoside or Eriodictyol pentoside	n.m.	18.65 ± 2.03	54.92 ± 1.59	n.d.	n.d.	n.d.	n.d.	n.d.	n.d.
^j^ Ellagic acid	n.d.	n.d.	n.d.	n.m.	n.d.	n.d.	n.m.	173.39 ± 12.47	259.21 ± 12.85
^i^ Resveratrol-*O*-glucoside (Piceid)	46.30 ± 3.15 a	28.52 ± 0.63 d	n.d.	141.92 ± 9.03 a	n.d.	n.d.	219.31 ± 22.65 a	105.66 ± 12.08 d	301.47 ± 24.61
^c^ Procyanidin trimer B-type isomer	n.d.	n.d.	n.d.	n.d.	n.m.	174.86 ± 0.66	n.d.	n.d.	n.d.
^g^ Dihydrokaempferol hexoside or Eriodictyol hexoside	71.64 ± 3.59	n.d.	n.d.	n.m.	n.d.	371.64 ± 42.16	n.d.	n.d.	n.d.
^g^ Isoquercitrin (quercetin-3-*O*-glucoside)	12.70 ± 3.38 a	n.m.	n.m.	204.62 ± 0.78 a	n.m.	227.82 ± 16.61	n.d.	n.d.	n.d.
^g^ Dihydrokaempferol hexoside or Eriodictyol hexoside	n.d.	n.d.	n.d.	150.86 ± 25.44	n.d.	190.54 ± 19.13	n.d.	n.d.	n.d.
^i^ Resveratrol dimer	51.80 ± 5.64	n.d.	n.d.	n.d.	n.d.	n.d.	n.d.	n.d.	n.d.
^g^ Quercetin 3-glucuronide	12.83 ± 1.38 a	n.m.	n.d.	194.33 ± 2.43 a	n.m.	42.98 ± 5.15	64.57 ± 6.29 a	n.d.	n.d.
^i^ Resveratrol dimer (caraphenol)	120.65 ± 7.16 a	14.93 ± 3.01 d	193.97 ± 2.16 f	248.50 ± 7.12 a,b	86.33 ± 21.56 d	501.89 ± 27.40 f,g	561.79 ± 29.03 b	187.09 ± 10.06 d	1050.47 ± 36.03 g
^g^ Naringenin-*O*-hexoside	132.93 ± 3.35 a	n.d.	n.d.	275.35 ± 2.87 a	n.d.	n.d.	165.61 ± 4.31 a	n.d.	n.d.
^c^ Procyanidin dimer A-type	n.d.	n.m.	140.89 ± 4.74	n.d.	n.d.	n.d.	n.d.	n.d.	n.d.
^i^ Resveratrol dimer	n.d.	n.d.	n.d.	n.d.	n.d.	n.d.	94.08 ± 14.40	n.d.	n.d.
^i^ Resveratrol	220.43 ± 16.84 a	51.51 ± 3.68 d	136.79 ± 43.31 f	746.78 ± 32.08 b	195.51 ± 15.99 d	679.61 ± 28.58 g	1741.01 ± 62.45 c	580.42 ± 0.33 e	1398.06 ± 23.58 h
^i^ Viniferol E	14.27 ± 1.31 a	5.29 ± 1.50 d	63.79 ± 9.69 f	15.76 ± 1.80 a	42.70 ± 2.72 d	38.82 ± 3.73 f	166.18 ± 7.53 b	191.56 ± 29.59 e	53.57 ± 2.44 f
^i^ Resveratrol tetramer	25.98 ± 8.90 a	n.d.	n.d.	73.78 ± 1.42 a	75.06 ± 3.00 d	72.11 ± 0.93 f	270.51 ± 1.86 a	232.36 ± 36.53 e	37.14 ± 2.22 f
^i^ ε-viniferin	16.42 ± 2.98 a	21.01 ± 3.69 d	23.83 ± 0.18 f	44.39 ± 1.73 a	40.12 ± 6.81 d	127.69 ± 6.76 g	1657.60 ± 18.80 b	1617.56 ± 199.55 e	1543.28 ± 97.67 h
^i^ Resveratrol tetramer	n.d.	n.d.	n.d.	n.d.	n.d.	n.d.	24.88 ± 6.80	n.d.	165.91 ± 2.14
^i^ Resveratrol dimer	n.d.	n.d.	n.d.	n.d.	n.d.	n.d.	169.04 ± 9.96	181.66 ± 16.13	n.m.

n.m. = not measurable. Compounds detected by HPLC-ITMS^n^ but not quantifiable by HPLC-UV; n.d. = not detected; ^a^ = compounds quantified using *p*-coumaric acid as reference; ^b^ = compounds quantified using gallic acid as reference; ^c^ = compounds quantified using catechin as reference; ^d^ = compounds quantified using protocatechuic acid as reference; ^e^ = compounds quantified using caffeic acid as reference; ^f^ = compounds quantified using syringic acid as reference; ^g^ = compounds quantified using quercetin as reference; ^h^ = compounds quantified using rosmarinic acid as reference; ^i^ = compounds quantified using resveratrol as reference; ^j^ = compounds quantified using ellagic acid as reference; ^k^ = compounds quantified using rutin as reference. Different letters in the same row denote significant differences at *p* < 0.05 (Aglianico: a-c; Fiano: d-e; Greco: f-h).

## Data Availability

The data presented in this study are available on request.

## Supplementary Materials

The following are available online. Figure S1, results of antibacterial tests on Gram negative (*E. coli*) and Gram positive (*S. aureus*) strains; Figure S2, results of antifungal tests on *C. albicans*.

## Sample Availability

Samples of the extracts used in the study are available from the authors.
