# Supplementary material for: Grape Canes from Typical Cultivars of Campania (Southern Italy) as a Source of High-Value Bioactive Compounds: Phenolic Profile, Antioxidant and Antimicrobial Activities
Source: Molecules. 2021 May 7;26(9):2746. doi: 10.3390/molecules26092746 (PMC8125794; doi:10.3390/molecules26092746)
Supplement: Supplementary file 1 [file molecules-26-02746-s001.zip › molecules-1196234-supplementary.pdf]

Grape canes from typical cultivars of Campania (Southern Italy) as a source of high-value bioactive compounds: phenolic profile, antioxidant and antimicrobial activities.

Giuseppe Squillaci[1,§], Carla Zannella [2,§], Virginia Carbone[3], Paola Minasi[3], Veronica Folliero[2], Debora Stelitano[2], Francesco La Cara[1], Massimiliano Galdiero[2], Gianluigi Franci[4,*], Alessandra Morana[1]

[1] Research Institute on terrestrial Ecosystems, National Research Council of Italy, via Pietro Castellino 111, 80131 Naples, Italy; giuseppe.squillaci@iret.cnr.it (G.S.); francesco.lacara@cnr.it (F.L.); alessandra.morana@cnr.it (A.M.)
[2] Department of Experimental Medicine, University of Campania "Luigi Vanvitelli", via Costantinopoli 16, 80138 Naples, Italy; carlazannella88@gmail.com (C.Z.); veronicafolliero88@gmail.com (V.F.); debora.stelitano@unicampania.it (D.S.); massimiliano.galdiero@unicampania.it (M.G.)
[3] Proteomic and Biomolecular Mass Spectrometry Center, Institute of Food Sciences, National Research Council of Italy, Via Roma 64, 83100 Avellino, Italy; virginia.carbone@cnr.it (V.C.); paola.minasi@cnr.it (P.M.)
[4] Department of Medicine, Surgery and Dentistry Scuola Medica Salernitana, University of Salerno, 84081 Salerno, Italy; gfranci@unisa.it (G.F.)
* Correspondence: gfranci@unisa.it; Tel.: +390815667569
§ G.S. and C.Z. equally contributed

Supplementary material

Figure S1, results of antibacterial tests on Gram negative *(E. coli*) and Gram positive (*S. aureus*) strains

Figure S2, results of antifungal tests on *C. albicans*

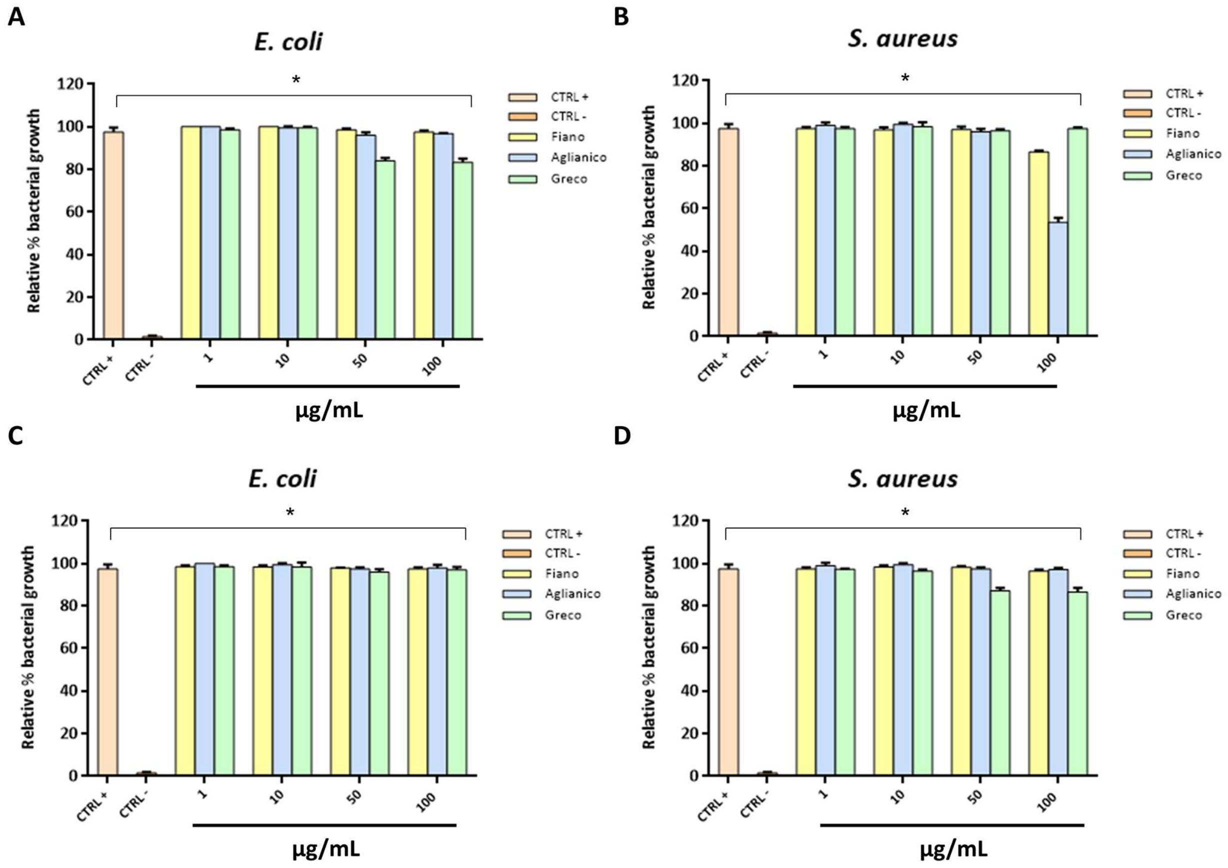

Figure S1. Antibacterial activity against gram-positive and gram-negative bacteria. *E. coli* and *S. aureus* growth was monitored after 20 hours of extracts at pH 7.00 (A and B) and 13.00 (C and D) addition. No considerable reduction of bacterial growth was observed, except for "Aglianico" pH 7.00 which inhibited S. aureus growth by 40% CTRL + referts to the positive control, that is the corresponding bacterial growth withouth any treatment; CTRL – corresponds to the negative control, that is *E.coli* treated with ampicillin (A and C) or *S. aureus* treated with vancomycin (B and D). Statistical differences were evaluated via Two-way ANOVA, a value of $p \leq 0.05$ was considered significant, with $^{*}p \leq 0.0001$.

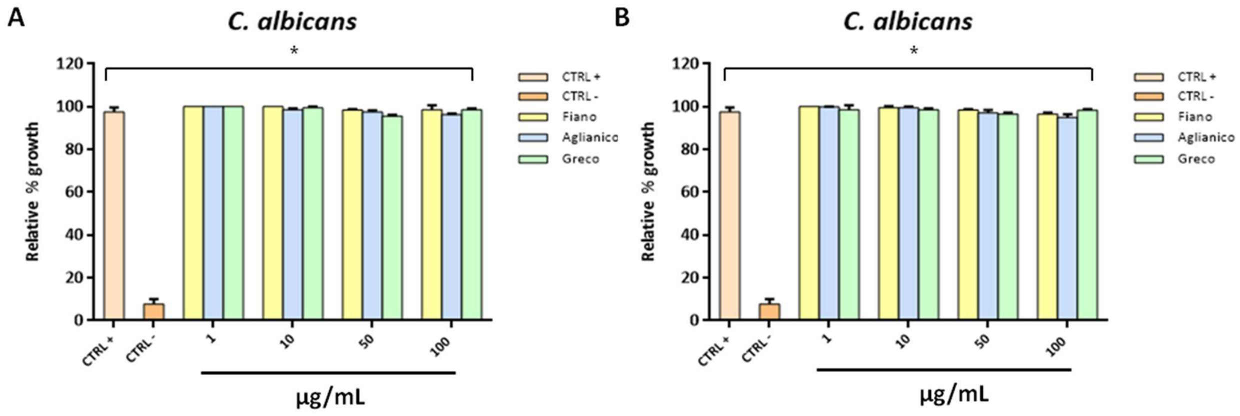

Figure S2: Antifungal activity against *C. albicans*. The growth was monitored after 48 hours of extracts at pH 7.00 (A) and 13.00 (B) addition. No considerable reduction of growth was observed. CTRL + referts to the positive control, that is only *C. albicans*; CTRL – corresponds to the negative control, that is *C. albicans* treated with fluconazole. Statistical differences were evaluated via Two-way ANOVA, a value of $p \leq 0.05$ was considered significant, with $^*p \leq 0.0001$.
